# Molecular Mechanisms of *CLCN5* Missense Mutations in Dent Disease Type 1: A Comprehensive Computational Analysis and Clinical Correlations in a Chinese Cohort

**DOI:** 10.1111/jcmm.71108

**Published:** 2026-03-20

**Authors:** Chengpeng Wu, Ying Zhang, Zipei Chen, Haidong Fu, Zhi Du, Liqun Chen, Guozhen Wang, Jianhua Mao, Lidan Hu

**Affiliations:** ^1^ Liangzhu Laboratory Zhejiang University School of Medicine Hangzhou China; ^2^ Eye Center of the Second Affiliated Hospital, Zhejiang University School of Medicine Hangzhou China; ^3^ Institute of Translational Medicine Zhejiang University School of Medicine Hangzhou China; ^4^ Department of Nephrology The Children's Hospital, Zhejiang University School of Medicine, National Clinical Research Center for Child Health Hangzhou China; ^5^ Department of Pharmacy Children's Hospital, Zhejiang University School of Medicine, National Clinical Research Center for Child Health Hangzhou Zhejiang China; ^6^ Academy of Medical Engineering and Translational Medicine Medical College, Tianjin University Tianjin China; ^7^ School of Basic Medical Sciences and Forensic Medicine Hangzhou Medical College Hangzhou Zhejiang China

**Keywords:** *CLCN5*, computational biology, dent's disease, genetic counselling, missense mutations, protein stability

## Abstract

Dent's disease, an X‐linked recessive disorder predominantly affecting males, is characterized by nephrocalcinosis, nephrolithiasis, and a high risk of progression to end‐stage renal disease. Dent's disease type 1, accounting for 60% of cases, caused by mutations in the *CLCN5* gene encoding the chloride ion channel protein ClC‐5, exhibits significant clinical heterogeneity and variability in disease progression. The lack of hotspot mutations poses challenges for genetic diagnosis and counselling, complicating the prediction of disease outcomes. This study systematically evaluated the functional and structural impacts of 181 *CLCN5* missense mutations using computational tools, including PredictSNP, MAGPIE, and molecular dynamics simulations, to propose a robust method for improving genetic counselling and prognosis prediction. Our analysis identified mutations at the dimer interface and chloride selectivity filter as critical disruptors of ClC‐5 function and stability. Notably, molecular dynamics simulations of L200R, P213L, and G512R mutations revealed that L200R significantly destabilized the protein structure. Clinical data from a multicentre cohort of Chinese patients with *CLCN5* mutations corroborated our computational predictions, highlighting the essential role of helix O in ClC‐5 function. By integrating bioinformatics analyses with clinical validation, this study provides molecular insights into Dent's disease heterogeneity and proposes a framework for enhancing genetic counselling and prognostic assessment for affected patients.

## 1 Introduction

Dent's disease is a rare X‐linked recessive disorder primarily affecting males and characterized by nephrocalcinosis, nephrolithiasis, and eventual progression to end‐stage renal disease within 30–50 years [1, 2, 3]. Clinically, it presents with low molecular weight proteinuria, hypercalciuria, renal calcification, and renal function abnormalities [4, 5]. Based on genetic and clinical features, Dent's disease is classified into three types, with Dent's disease 1 accounting for 60% of cases and being the most common, caused by mutations in the *CLCN5* gene encoding the chloride ion channel ClC‐5 [6, 7]. Despite advances in understanding the genetic basis, the variability in clinical outcomes and the lack of hotspot mutations complicate genetic diagnosis and counselling, creating challenges for predicting disease progression and providing precise therapeutic guidance [8].

The *CLCN5* gene, located on the X chromosome at position Xp11.23, encodes a protein crucial for endosomal acidification and albumin uptake in proximal renal tubules. The structural integrity and function of the ClC–5 protein are hypothesized to rely on its predicted dimeric architecture and its role as a chloride/proton exchanger [9, 10, 11, 12]. Each subunit of ClC‐5 contains two phosphorylation sites and one N‐glycosylation site, with N‐glycosylation playing a key role in protein folding and oligomerization [13, 14]. Defective N‐glycosylated proteins are retained in the endoplasmic reticulum, while correctly glycosylated proteins are targeted to the plasma membrane and early endosomes [15]. The carboxy terminus of ClC‐5 includes two CBS domains, a PY motif, and a PDZ binding domain, with multiple potential regulatory signals for protein interactions involved in endocytic processes [16, 17]. Proper folding at the C‐terminus is essential for endoplasmic reticulum quality control, and mutations in this region can lead to protein retention and reduced activity [8]. ClC‐5 is involved in endosome acidification and albumin uptake in the proximal renal tubules, co‐localizing with V‐type ATPase [18, 19]. The endocytosis of albumin is a coordinated process involving proteins such as megalin, cubilin, V‐H+ ATPase, NHE3, and the Cl‐ channel ClC‐5 [20]. Actin depolymerization by cofilin may participate in ClC‐5 endocytosis, with cofilin phosphorylation by LIM kinase 1 maintaining actin stability [21]. In proximal tubule cell cultures, cofilin co‐localizes with ClC‐5, and its phosphorylation is inhibited during albumin uptake, suggesting a role in endosome transport [21]. Missense mutations in *CLCN5* can disrupt key regions of the protein, such as the dimer interface, chloride selectivity filter, and CBS domains, leading to impaired protein function, intracellular degradation, and disease manifestation [22, 23]. Understanding how these mutations affect the protein at a molecular level is essential to developing predictive models for clinical outcomes and guiding genetic counselling.

This study systematically investigates the molecular mechanisms underlying 181 *CLCN5* missense mutations using bioinformatics tools and molecular dynamics simulations. Predictive computational analyses were corroborated with preliminary clinical data from a multicentre cohort of Chinese patients, providing a preliminary basis for future genetic counselling and prognostic studies—with further validation required in larger, multi‐ethnic cohorts. The analysis focused on identifying mutations that disrupt critical structural and functional regions of ClC‐5, particularly those contributing to disease heterogeneity and progression variability.

By integrating computational predictions with clinical validation, this work proposes a systematic approach to address the challenges of genetic counselling and disease outcome prediction in Dent's disease. The findings offer novel insights into the molecular basis of the disease and identify key regions of ClC‐5 as potential therapeutic targets, paving the way for personalized interventions.

## 2 Materials and Methods

### 2.1 Online Data Retrieval

We sourced the standard *CLCN5* protein sequence from the National Library of Medicine (NCBI Reference Sequence: NP_000075.1). Missense mutations were compiled from UniProt (UniProt ID: P51795), the Human Gene Mutation Database (HGMD, http://www.hgmd.org/), and ClinVar databases (https://www.ncbi.nlm.nih.gov/clinvar/). Protein and mutation data were also obtained through the Online Mendelian Inheritance in Man (OMIM, https://omim.org/) database.

Employing the keyword “*CLCN5*,” we conducted a thorough search across HGMD, UniProt, and ClinVar databases to identify clinically relevant missense mutations. We selected mutations associated with *CLCN5*, adhering to ACMG guidelines for experimental and clinical evidence, and classified them as pathogenic or likely pathogenic [22, 24, 25, 26, 27].

### 2.2 Pathogenicity Prediction of Mutations

The PredictSNP2 tool (https://loschmidt.chemi.muni.cz/predictsnp/) and the Multimodal Annotation Generated Pathogenic Impact Evaluator (MAGPIE, http://tools.shenlab‐genomics.org/tools/MAGPIE) analyzer were utilized to assess the functional impact of mutations. PredictSNP integrates various algorithms, including MAPP, PhD‐SNP, PolyPhen1, PolyPhen2, SIFT, SNAP, PANTHER, and nsSNP [28]. MAGPIE scores range from 0 to 1, with higher scores indicating increased pathogenicity [29]. The *CLCN5* mRNA sequence (NM_000084.5) was retrieved from NCBI (https://ncbi.nlm.nih.gov/nuccore/NM_000084.5). Default parameters were applied for mutation analysis, and results were interpreted based on PredictSNP (score > 75) and MAGPIE (score > 0.5) criteria.

### 2.3 Stability Prediction of Mutations

The iStable server (http://predictor.nchu.edu.tw/istable/), incorporating iMutant and MUpro, predicts protein stability changes using thermodynamic parameters [30]. iMutant calculates free energy differences (ΔΔG), while MUpro provides a confidence score for stability effects. We input mutated sequences into iStable for thermodynamic parameter predictions and recorded ΔΔG values to assess stability impacts.

### 2.4 Align GVGD

Align GVGD (http://agvgd.hci.utah.edu/agvgd_input.php) combines MSA with amino acid biophysical properties to evaluate the impact of nsSNPs, classifying them from benign to harmful [31]. We input sequences and mutations to obtain classifications from class 15 (neutral) to class 65 (deleterious). Deleterious mutants were further analyzed for evolutionary conservation using the ConSurf server.

### 2.5 SNPeffect

The human SNP effect server (https://snpeffect.switchlab.org/), designed for phenotyping, annotates variants in the human proteome and characterizes diseases and polymorphisms at the molecular level [32]. It integrates TANGO, WALTZ, LIMBO, and FoldX for a comprehensive analysis of SNP effects.

### 2.6 Prediction of the ClC‐5 Protein Structure

We used AlphaFold3 (https://alphafoldserver.com/) to predict wild‐type ClC‐5 protein structures [33]. Utilize PyMOL software to perform structural alignment of the predicted wild‐type and mutant protein structures. Create visualizations and generate illustrative images of structural alignments using PyMOL or ChinmeraX, highlighting the mutation sites and their impact on the surrounding structure. MOLE2 predicts the membrane region of ClC‐5 [34].

### 2.7 Molecular Dynamics Simulations of Wild‐Type and Mutant ClC‐5 Proteins

Wild‐type and mutant ClC‐5 proteins were subjected to GROMACS‐based molecular dynamics simulations to study structural and dynamic changes (https://www.gromacs.org/). The atomic coordinates of the wild‐type ClC‐5 protein were retrieved from Prediction of the ClC‐5 protein structure. Mutagenesis was performed on the wild‐type structure to introduce the L200R, P213L, and G512R mutations using the Swiss‐PdbViewer tool (https://spdbv.unil.ch/), which allows for the modification of amino acid residues in the protein structure. Proteins were solvated in a cubic box, neutralized with counter‐ions, and the GROMOS53A6 force field and SPC water model were applied. The system underwent energy minimization using the steepest descent algorithm. Equilibration occurred in NVT and NPT ensembles, allowing solvent and system equilibrium. The production run used a 2 fs time step, controlled temperature at 310 K, and pressure at 1 bar, lasting 100 ns. MD trajectories were analyzed for stability and dynamics, including RMSD, RMSF, radius of gyration, hydrogen bonding, SASA, and secondary structure content.

### 2.8 Clinical Information

Clinical data were collected from a cohort of 85 patients with Dent‐I disease from 2014 to 2025, established through the Chinese Children Genetic Kidney Disease Database (CCGKDD, www.ccgkdd.com.cn). The study was approved by the Children's Hospital of Zhejiang University School of Medicine (2021‐IRB‐092). Among the 22 *CLCN5* missense mutations identified in patients with Dent disease type 1, most have been documented in public databases. However, the mutations G250D, G260D, T277P, L319R, M504V, and V522F have not been recorded in HGMD, UniProt, or ClinVar. Notably, G250D and T277P have been previously reported by our group [35].

## 3 Results

### 3.1 In Silico Analysis of Mutation Impact

181 missense mutations of *CLCN5* were sourced from the UniProt, HGMD, and ClinVar databases (Table S1). Using PredictSNP, the missense mutations were analyzed and categorized primarily into deleterious or neutral categories. Among the other computational tools, PredictSNP, MAPP, PhD‐SNP, PolyPhen‐1, PolyPhen‐2, SIFT, SNAP, and PANTHER designated 111, 93, 116, 109, 113, 129, 94, and 21 as deleterious, respectively. Meanwhile, 93 missense mutations were classified as deleterious by at least 6 bioinformatics tools. (Table S2). On the MAGPIE website, there are 101 missense mutations with a score greater than 0.72, which may be considered potential pathogenic sites (Table S3). The iStable server, utilizing iMutant, MUpro, and its own assessments, predicted a decrease in protein stability for a significant number of mutations (Table S4).

### 3.2 AlphaFold3‐Predicted Structure of the Dimeric ClC‐5

**FIGURE 1:**
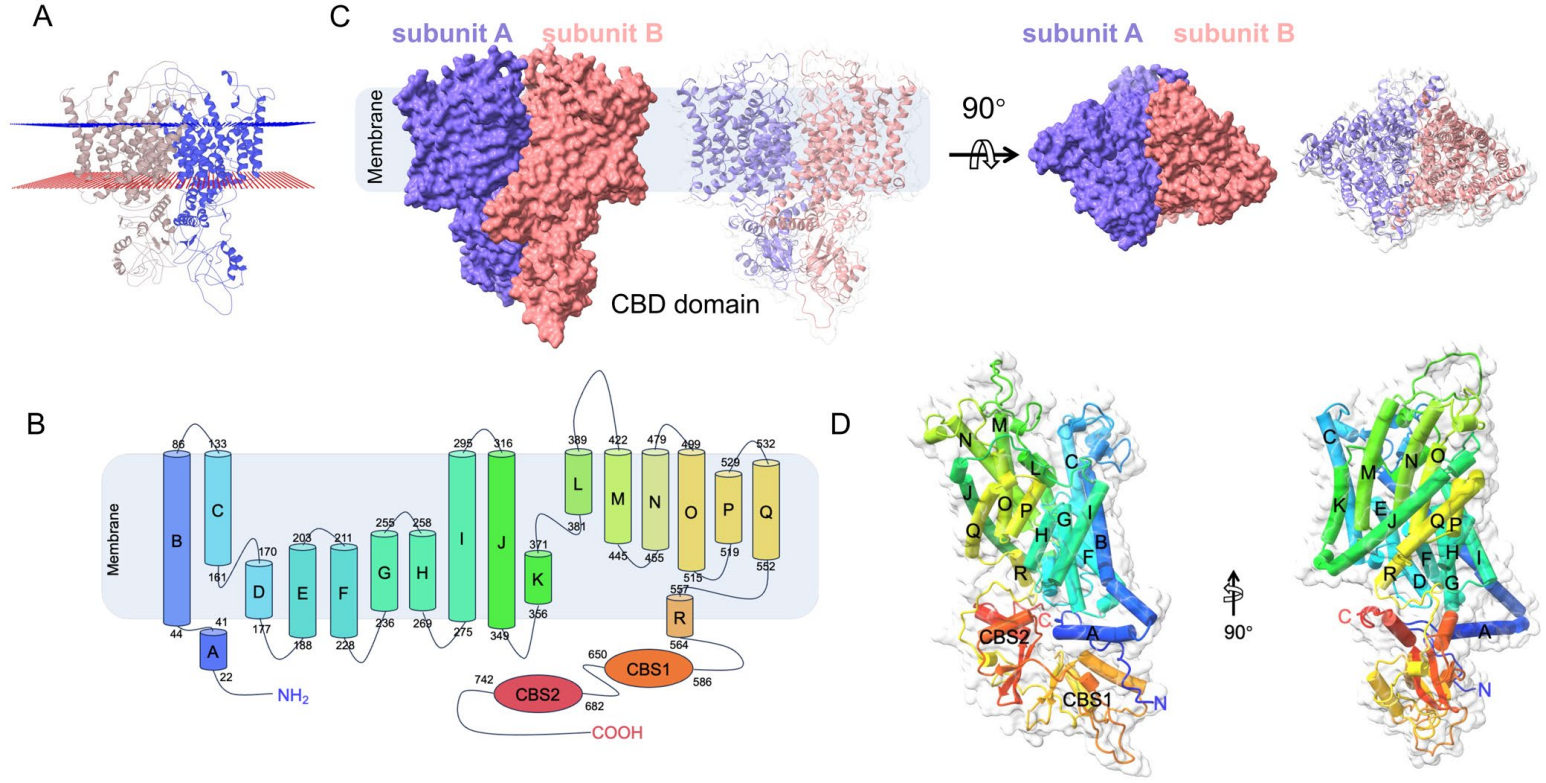
Structural representation of the predicted CLC‐5 dimer. (A) MOLE2 predicts the membrane region of ClC‐5. (B) Topology and domain arrangement of ClC‐5. (C) Cartoon representation of ClC‐5 with each subunit individually coloured. (D) The structure of ClC‐5 subunit A.

We employed AlphaFold3 to predict the dimeric structure of the ClC‐5 protein, yielding a model that aligns with previously resolved ClC protein structures. Our analysis revealed that one subunit of the predicted dimeric ClC‐5 possesses 18 alpha‐helices, with 17 of these (Helix B to Helix R) spanning the transmembrane domain (Figure 1A,B). Consistent with other ClC exchangers, the adenosine nucleotide‐binding cystathionine beta‐synthase (CBS) domain is located on the cytoplasmic side (Figure 1A,B).

Notably, the dimeric interface of ClC‐5 is formed by helices B, H, I, P, and Q. Through our structural predictions (Figure 1C,D), we have identified the amino acid residues at the subunit interfaces that mediate inter‐subunit interactions within the dimer (Table S5). These residues are crucial for the assembly and function of the ClC‐5 dimer, providing insights into its potential role in chloride transport and ion channel regulation.

### 3.3 Missense Mutations Across Different Structural Domains

**FIGURE 2:**
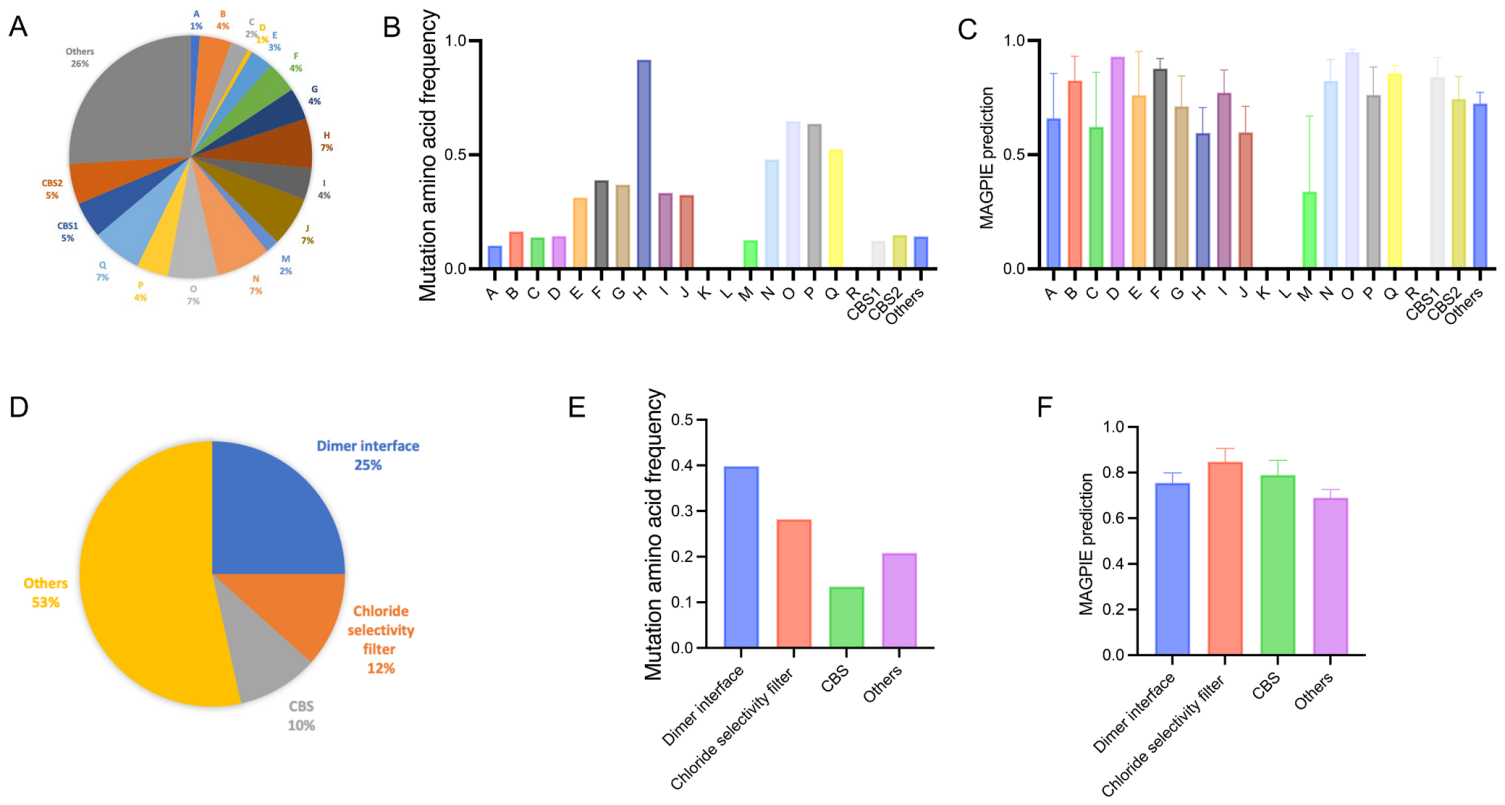
Distribution and frequency of missense mutations across different structural domains of ClC‐5 protein. (A) The proportion of mutations across different structural domains out of all mutations. Others refers to amino acid mutation sites other than structural domains. (B) The frequency of mutations occurring across different structural domains. (C) The MAGPIE prediction pathogenic scores for mutations across different structural domains. (D) The proportion of mutations across different functional regions out of all mutations. Others refers to amino acid mutation sites other than different functional regions. (E) The frequency of mutations occurring across different functional regions. (F) The MAGPIE prediction pathogenic scores for mutations across different functional regions.

We initiated our analysis by comparing the proportion of missense mutations across various domains, including the 18 alpha‐helices, CBS1, CBS2, and other regions. Notably, aside from the ‘others’ category, mutations were most frequently observed in helices H, J, N, O, and Q (Figure 2A). Further analysis of the frequency of missense mutations within these domains revealed that helices H, O, P, and Q exhibited the highest mutation rates (Figure 2B). Subsequently, we assessed the pathogenic scores associated with mutations in these domains (Figure 2C). Helix O demonstrated the highest average score, underscoring its critical role in the structure and function of the ClC‐5 protein.

We then categorized the domains based on their functional roles: Dimer interface (helices B, H, I, P, Q), Chloride selectivity filter (helices D, N, F, R) [36], CBS (CBS1, CBS2), and Others. Among these, the Dimer interface had the highest proportion of missense mutations (Figure 2D), and also the highest mutation frequency (Figure 2E), excluding the ‘Others’ category. Interestingly, the chloride selectivity filter showed the highest average pathogenic score, and all three functional regions had average scores higher than those of the ‘Others’ category (Figure 2E). These findings suggest that the Dimer interface and Chloride selectivity filter are particularly sensitive to missense mutations, which may have significant implications for the protein's function and stability.

### 3.4 Biophysical and Conservation Evaluation

**FIGURE 3:**
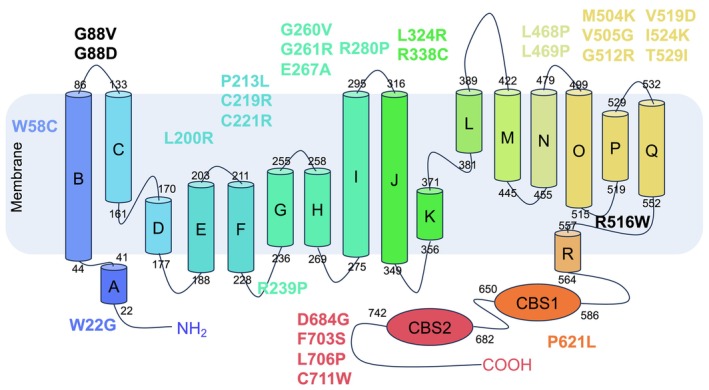
The distribution of the preferred candidate mutations across structural domains.

The biophysical impact of mutations was assessed through multiple sequence alignment using the align GVGD tools, which assigned a GV score and classified all mutations into a class, indicating a high likelihood of deleterious impact (Table S6). According to the outputs from all the tools, W22G, W58C, G88D, G88V, L200R, P213L, C219R, C221R, R239P, G260V, G261R, E267A, R280P, L324R, R338C, L468P, L469P, M504K, V505G, G512R, G512V, R516W, V519D, I524K, T529I, P621L, D684G, F703S, L706P and C711W were identified as the preferred candidate mutations for this study (Figure 3). All 30 were re‐predicted by the abovementioned tools; the results indicated that they were consistently deleterious and tended to decrease protein stability.

### 3.5 SNPeffect

The findings from the SNPeffect database indicated that the G88V, P213L, G260V, G261R, E267A, R338C, and R516W mutations increase the aggregation tendency of the protein, while the L200R, L324R, M504K, and V505G mutations decrease the aggregation tendency of the protein. Meanwhile, G260V, G261R, and T529I were found to reduce the amyloid propensity of the protein, while R516W, V519D, and I524K were shown to enhance it. Additionally, none of the 30 variants were found to affect the protein's ability to bind chaperones (Table S7).

### 3.6 The Molecular Dynamics Simulations of the L200R, P213L, and G512R Mutations

**FIGURE 4:**
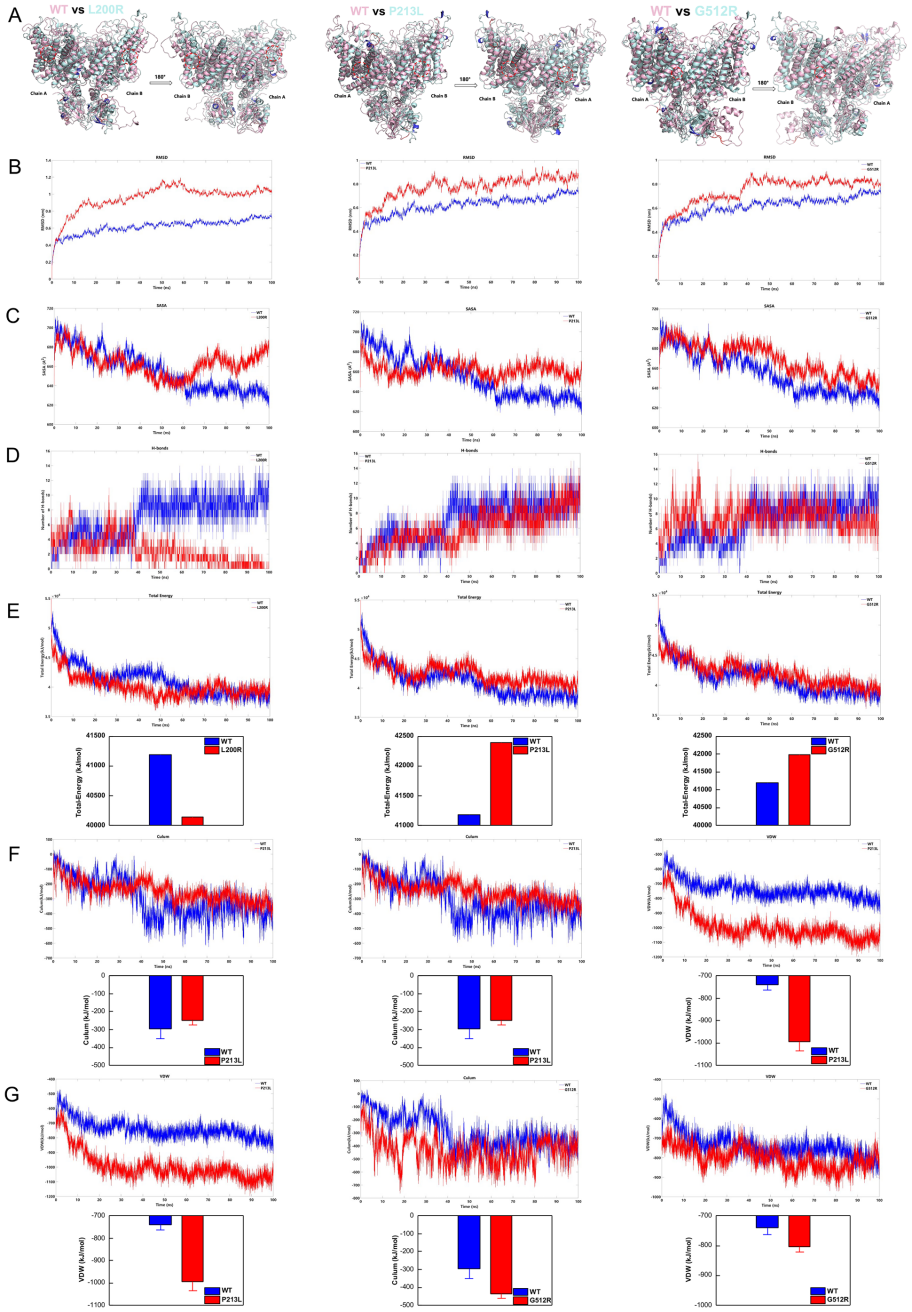
Molecular dynamics simulation results for L200R, P213L, and G512R mutations. (A) Structural changes of L200R, P213L, and G512R after 100 ns of molecular dynamics simulation. (B–D) Changes in RMSD, SASA, and hydrogen bond numbers during the molecular dynamics simulation process. (E–G) Changes in the total energy, Coulombic force, and van der Waals forces of the protein during the molecular dynamics simulation process.

To systematically investigate how missense mutations affect protein stability through different pathogenic mechanisms, we selected three representative mutations. L200R was chosen because it reduced the protein's aggregation propensity in previous analyses, while P213L increased the protein's aggregation propensity. G512R was selected because it does not affect the protein's aggregation propensity; however, it is located in the o‐helix and may thus impact protein function. We conducted molecular dynamics simulations to compare the structural changes in the protein after the L200R, P213L, and G512R mutations (Figure 4A). The root mean square deviation (RMSD) results indicated that all three mutations rendered the protein structure less stable compared to the wild type, with the L200R mutation exerting the most destabilizing effect (Figure 4B).

The solvent‐accessible surface area (SASA) analysis revealed that all mutations increased the protein's accessible surface area, with the L200R mutation showing the most significant increase (Figure 4C). Hydrogen bonding statistics demonstrated a decrease in the number of hydrogen bonds for the L200R mutation relative to the wild type (Figure 4D).

Energy analysis showed that the total energy of the L200R mutation decreased compared to the wild type, while the total energy increased for both the P213L and G512R mutations (Figure 4E). For the L200R mutation, both Coulombic and van der Waals forces decreased, with the Coulombic force showing the most pronounced change (Figure 4F,G). In contrast, the G512R mutation resulted in increased Coulombic and van der Waals forces, while the P213L mutation showed a decrease in Coulombic force and a significant increase in van der Waals force (Figure 4F,G).

These results suggest that the L200R mutation has the most significant impact on protein structure, while the G512R mutation has a comparatively minor effect.

### 3.7 Comparison Between the Clinical Patient Data of *CLCN5* Gene Mutations in China and Predicted Results

**FIGURE 5:**
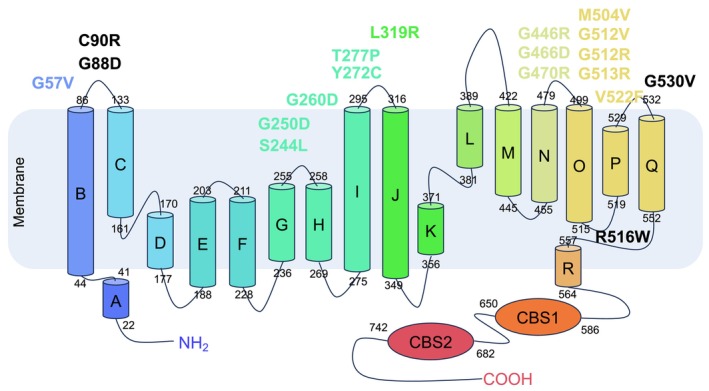
The distribution of clinical mutations across structural domains.

We collected clinical data from 85 Chinese patients with Dent disease type 1 who carry *CLCN5* gene mutations, among whom 22 have *CLCN5* missense mutations (Table S8). These mutations were compared with our prediction results (Table S9). Of the 22 patients, 5 (22.7%) carry missense mutations located in the O‐helix, which further underscores the critical role of the O‐helix in the ClC‐5 protein (Figure 5). The 22 patients harbour 19 distinct missense mutations, 17 of which (89.4%) were classified as deleterious by at least 8 out of 10 tools in previous predictions, demonstrating the effectiveness of our integrated prediction approach. In contrast, G57V was classified as deleterious by only 4 tools, and M504V by only 3 tools. These findings highlight the importance of our prediction model in identifying mutations that may contribute to clinical manifestations, and provide valuable insights into the functional domains essential for the normal function of the ClC‐5 protein.

## 4 Discussion

This study systematically analyzed the impacts of 181 missense mutations in the *CLCN5* gene to improve the genetic diagnosis and counselling for Dent's disease type 1. By utilizing state‐of‐the‐art computational tools and integrating clinical data from a multicentre cohort of Chinese patients, we propose a framework for predicting the pathogenicity and clinical relevance of these mutations. The findings aimed to address the challenges posed by the clinical heterogeneity of Dent's disease and the lack of hotspot mutations, which have complicated traditional diagnostic and counselling approaches.

Our in silico analysis utilizing tools like PredictSNP and MAGPIE revealed a significant number of deleterious mutations, with a subset showing a substantial decrease in protein stability as predicted by iStable. These findings underscore the importance of specific residues in maintaining the structural integrity and function of ClC‐5, particularly within the dimer interface and chloride selectivity filter. The high mutation rates and pathogenic scores observed in these regions suggest a heightened sensitivity to missense mutations, which may disrupt the protein's normal function and contribute to the disease phenotype. Alterations in the functions of the ClC‐5 ion channels may affect endosomal acidification, leading to endocytic defects in the proximal renal tubules and triggering Dent disease [37, 38]. Mutations at the dimer interface of the ClC‐5 protein can affect helix stability, disrupt protein formation, and lead to rapid intracellular degradation, as well as impair the channel's structural integrity and function [39].

The biophysical evaluation using align GVGD and SNPeffect further refined our candidate mutations, identifying a set of mutations that are likely to destabilize the ClC‐5 protein and affect its aggregation propensity. Notably, mutations such as L200R, P213L, and G512R were selected for molecular dynamics simulations. These simulations confirmed that the L200R mutation, in particular, rendered the protein structure less stable, with a decrease in hydrogen bonding and an increase in solvent‐accessible surface area, consistent with a loss of function. The L200R mutation included in this study has previously been identified in Dent disease patients and reported to reduce chloride ion currents, while our findings further complement its underlying mechanism [40].

The clinical correlation of our predicted mutations with patient data from China provided a validation step for our computational approach. Among the 19 *CLCN5* missense mutations identified in our patient cohort, 17 were predicted as deleterious by at least 8 bioinformatics tools, which demonstrates the effectiveness of our integrated prediction approach. The identification of mutations in helix O in 5 patients further emphasizes the functional importance of this region in ClC‐5, as suggested by our structural analysis. In Italian patients with Dent's disease 1, the G512D mutation in the *CLCN5* gene has also been identified [41]. In a retrospective study of 162 Dent disease type 1 (DD1) patients encompassing the United States, Italy, and South Korea, G88D, C90R, S244L, Y272C, G366D, and R516W—mutations also present in our cohort—have been reported [42]. As a member of the ClC family of exchangers, ClC‐3 is homologous to ClC‐4 and ClC‐5 and is widely expressed in the membranes of endosomes [13, 43]. In the resolved structure of ClC‐3, helix O plays a significant role in the chloride ion transport pathway [44]. The loss or reduction of chloride channel function due to *CLCN5* gene mutations is the primary pathological basis of Dent's disease [45]. This suggests that the G512R/V/D mutation may be causing disease by affecting chloride ion transport through the chloride ion channel.

From the perspective of molecular dynamics simulations, for the P213L mutation, results show changes in the protein's total energy, as well as alterations in Coulombic and van der Waals forces. These changes in energy and forces can affect the intra‐ and intermolecular interactions of the protein. It may disrupt the binding ability of the protein to other related proteins or influence its localization and stability in the cell membrane, thus interfering with the normal transport of chloride ions [46].

As for the L200R mutation, molecular dynamics simulations demonstrate a significant impact on the protein structure. It leads to an increase in the root‐mean‐square deviation (RMSD), an increase in the solvent‐accessible surface area, and a decrease in the number of hydrogen bonds. These structural changes are likely to disrupt the normal folding and conformation of the ClC–5 protein, preventing it from forming an effective chloride channel or affecting the opening and closing function of the channel. Consequently, the transmembrane transport of chloride ions is affected, contributing to the manifestation of disease symptoms. Studies on the functional consequences of *CLCN5* mutations in heterologous expression systems have shown that mutations frequently lead to protein folding and processing defects, causing the misfolded ClC‐5 proteins to be retained in the endoplasmic reticulum [37, 45, 47]. The misfolded ClC‐5 mutants may induce endoplasmic reticulum (ER) stress, resulting in oxidative stress and damage to proximal tubule cells [48, 49]. A mouse model carrying the N340K pathogenic mutation was established to study the consequences of misfolded ClC‐5 intracellular retention, and these mice exhibit ClC‐5 intracellular retention in proximal tubular cells along with increased urinary calcium and glucose excretion, decreased urinary pH, and severe low‐molecular‐weight proteinuria, features that summarize the common characteristics of Dent Disease [50].

Despite these contributions, this study has several limitations. On the one hand, the research relied solely on computational predictions to evaluate the impact of *CLCN5* mutations. While these methods provide a high‐throughput and cost‐effective approach for identifying pathogenic mutations, they do not directly elucidate the molecular mechanisms by which these mutations disrupt protein function. Experimental systems, such as site‐directed mutagenesis, electrophysiological assays, or endosomal acidification studies, are necessary to validate and expand upon these findings. On the other hand, while the integration of clinical data from a multicentre cohort strengthens the study's relevance, the dataset is limited to Chinese patients and may not fully represent the global genetic diversity of Dent's disease. Expanding this framework to include diverse populations could enhance its generalizability and provide additional insights into mutation‐specific disease manifestations.

In conclusion, our study provides a comprehensive evaluation of the impact of missense mutations in the *CLCN5* gene on the structure and function of the ClC‐5 protein. The integration of computational predictions, biophysical assessments, and clinical data offers a robust framework for understanding the molecular basis of Dent's disease 1. Our findings not only advance the understanding of the disease's pathogenesis but also offer potential therapeutic targets for intervention. Future studies should focus on experimental validation of the predicted effects of these mutations and the development of targeted therapies to mitigate the progression of Dent's disease 1.

## Author Contributions

**Chengpeng Wu:** conceptualization (equal), investigation (equal), methodology (equal), software (equal), validation (equal), visualization (equal), writing – original draft (equal), writing – review and editing (equal). **Ying Zhang:** conceptualization (equal), investigation (equal), methodology (equal), validation (equal), writing – original draft (equal), writing – review and editing (equal). **Zipei Chen:** validation (equal), visualization (equal), writing – original draft (equal), writing – review and editing (equal). **Haidong Fu:** data curation (equal), formal analysis (equal), resources (equal), validation (equal), writing – original draft (equal). **Zhi Du:** investigation (equal), validation (equal), writing – original draft (equal), writing – review and editing (equal). **Liqun Chen:** investigation (equal), visualization (equal), writing – original draft (equal). **Guozhen Wang:** data curation (equal), software (equal), validation (equal). **Jianhua Mao:** supervision (equal), validation (equal), writing – review and editing (lead). **Lidan Hu:** conceptualization (equal), formal analysis (equal), funding acquisition (lead), investigation (equal), methodology (equal), supervision (equal), writing – original draft (equal), writing – review and editing (equal).

## Funding

Natural Science Foundation of Zhejiang Province, No. R25H120005, National Natural Science Foundation of China, No. 82200784.

## Conflicts of Interest

The authors declare no conflicts of interest.

## Supporting information


**Table S1:** List of CLCN5 missense mutations retrieved from UniProt, HGMD, and ClinVar databases.
**Table S2:** Deleteriousness prediction of CLCN5 mutations using the PredictSNP server.
**Table S3:** Predict the pathogenicity of CLCN5(NM_000084.5) mutations using the MAGPIE website.
**Table S4:** Change in stability prediction of CLCN5 mutations using the iStable server.
**Table S5:** Dimer interface interactions.
**Table S6:** Biophysical characterization of CLCN5 mutations using the Align GVGD server.
**Table S7:** Phenotypic effect prediction of pathogenic CLCN5 mutations using the SNPeffect server.
**Table S8:** Clinical patient data from individuals with CLCN5 gene mutations in China.
**Table S9:** Pathogenicity predictions of clinical patient with CLCN5 gene mutations in China.

## Data Availability

Data available in article Supporting Information.
