# Supplementary material for: Molecular Mechanisms of CLCN5 Missense Mutations in Dent Disease Type 1: A Comprehensive Computational Analysis and Clinical Correlations in a Chinese Cohort
Source: J Cell Mol Med. 2026 Mar 20;30(6):e71108. doi: 10.1111/jcmm.71108 (PMC13097397; doi:10.1111/jcmm.71108)
Supplement: Supplementary file 1 — Table S1: List of CLCN5 missense mutations retrieved from UniProt, HGMD, and ClinVar databases. Table S2: Deleteriousness prediction of CLCN5 mutations using the PredictSNP server. Table S3: Predict the pathogenicity of CLCN5(NM_000084.5) mutations using the MAGPIE website. Table S4: Change in stability prediction of CLCN5 mutations using the iStable server. Table S5: Dimer interface interactions. Table S6: Biophysical characterization of CLCN5 mutations using the Align GVGD server. Table S7: Phenotypic effect prediction of pathogenic CLCN5 mutations using the SNPeffect server. Table S8: Clinical patient data from individuals with CLCN5 gene mutations in China. Table S9: Pathogenicity predictions of clinical patient with CLCN5 gene mutations in China. [file JCMM-30-e71108-s001.docx]

Table S1. List of CLCN5 missense mutations retrieved from UniProt, HGMD, and ClinVar databases.

| S.No | Accession number | Amino acid change |
| --- | --- | --- |
| 1. | CM983857 NM_000084.5:c.64T>G | W22G |
| 2. | CM187918 NM_000084.5:c.88C>T | R30W |
| 3. | CM1617184 NM_000084.5:c.169G>C | G57R |
| 4. | CM970315 NM_000084.5:c.169G>T | G57V |
| 5. | CM097568 NM_000084.5:c.174G>C | W58C |
| 6. | CM095944 NM_000084.5:c.173G>T | W58L |
| 7. | CM1314273 NM_000084.5:c.193G>A | G65R |
| 8. | NM_000084.5:c.238C>A | H80N |
| 9. | CM066011 NM_000084.5:c.263G>A | G88D |
| 10. | CM139666 NM_000084.5:c.263G>T | G88V |
| 11. | CM139667 NM_000084.5:c.268T>C | C90R |
| 12. | CM139667 NM_000084.5:c.270C>G | C90W |
| 13. | CM1414749 NM_000084.5:c.299A>G | H100R |
| 14. | CM082570 NM_000084.5:c.302G>A | C101Y |
| 15. | CM139669 NM_000084.5:c.307T>C | W103R |
| 16. | NM_000084.5:c.344A>G | K115R |
| 17. | NM_000084.5:c.373C>G | L125V |
| 18. | CM1414345 NM_000084.5:c.389A>G | D130G |
| 19. | NM_000084.5:c.426G>A | M142I |
| 20. | NM_000084.5:c.472G>C | V158L |
| 21. | CM139671 NM_000084.5:c.527T>A | I176N |
| 22. | CM097071 NM_000084.5:c.536G>A | G179D |
| 23. | NM_000084.5:c.586A>G | I196V |
| 24. | CM960308 NM_000084.5:c.599T>G | L200R |
| 25. | CM097072 NM_000084.5:c.608C>T | S203L |
| 26. | CM139673 NM_000084.5:c.631G>C | E211Q |
| 27. | CM139674 NM_000084.5:c.634G>A | G212S |
| 28. | CM097073 NM_000084.5:c.635G>C | G212A |
| 29. | CM139675 NM_000084.5:c.638C>T | P213L |
| 30. | CM071612 NM_000084.5:c.655T>C | C219R |
| 31. | CM044214 NM_000084.5:c.661T>C | C221R |
| 32. | CM077238 NM_000084.5:c.674T>C | L225P |
| 33. | CM157776 NM_000084.5:c.692A>T | K231I |
| 34. | CM157777 NM_000084.5:c.716G>C | R239P |
| 35. | NM_000084.5:c.716G>A | R239H |
| 36. | NM_000084.5:c.715C>T | R239C |
| 37. | CM960309 NM_000084.5:c.731C>T | S244L |
| 38. | NM_000084.5:c.746C>T | A249V |
| 39. | CM157778 NM_000084.5:c.748G>C | G250R |
| 40. | NM_000084.5:c.749G>A | G250D |
| 41. | NM_000084.5:c.751G>A | V251I |
| 42. | NM_000084.5:c.766G>C | G256R |
| 43. | NM_000084.5:c.779G>A | G260D |
| 44. | CM062520 NM_000084.5:c.779G>T | G260V |
| 45. | CM095945 NM_000084.5:c.781G>A | G261R |
| 46. | CM104899 NM_000084.5:c.782G>A | G261E |
| 47. | CM166359 NM_000084.5:c.789A>T | L263F |
| 48. | NM_000084.5:c.794G>T | S265L |
| 49. | CM166360 NM_000084.5:c.793A>C | S265R |
| 50. | CM139676 NM_000084.5:c.796C>G | L266V |
| 51. | CM153737 NM_000084.5:c.799G>A | E267K |
| 52. | CM157779 NM_000084.5:c.801A>C | E267D |
| 53. | CM044216 NM_000084.5:c.800A>C | E267A |
| 54. | CM185956 NM_000084.5:c.800A>T | E267V |
| 55. | CM1516353 NM_000084.5:c.804G>C | E268D |
| 56. | CM157783 NM_000084.5:c.810C>G | S270R |
| 57. | CM980369 NM_000084.5:c.808A>C | S270R |
| 58. | CM044217 NM_000084.5:c.808A>G | S270G |
| 59. | CM139677 NM_000084.5:c.814T>A | Y272N |
| 60. | CM066756 NM_000084.5:c.815A>G | Y272C |
| 61. | CM071613 NM_000084.5:c.817T>C | F273L |
| 62. | NM_000084.5:c.829A>C | T277P |
| 63. | CM980370 NM_000084.5:c.834G>C | L278F |
| 64. | CM166356 NM_000084.5:c.833T>C | L278S |
| 65. | CM122880 NM_000084.5:c.833T>G | L278W |
| 66. | CM970316 NM_000084.5:c.839G>C | R280P |
| 67. | NM_000084.5:c.851C>T | A284V |
| 68. | NM_000084.5:c.877C>T | R293C |
| 69. | NM_000084.5:c.898A>G | N300D |
| 70. | NM_000084.5:c.905G>A | R302H |
| 71. | NM_000084.5:c.904C>T | R302C |
| 72. | NM_000084.5:c.931C>T | H311Y |
| 73. | NM_000084.5:c.935C>A | T312N |
| 74. | NM_000084.5:c.940T>C | W314R |
| 75. | NM_000084.5:c.956T>G | L319R |
| 76. | CM052170 NM_000084.5:c.971T>G | L324R |
| 77. | CM122879 NM_000084.5:c.986G>A | G329D |
| 78. | CM077523 NM_000084.5:c.997G>A | G333R |
| 79. | NM_000084.5:c.1013G>A | R338H |
| 80. | NM_000084.5:c.1012C>T | R338C |
| 81. | CM066755 NM_000084.5:c.1020C>G | N340K |
| 82. | CM165162 NM_000084.5:c.1033C>T | R345W |
| 83. | NM_000084.5:c.1034G>T | R345L |
| 84. | NM_000084.5:c.1034G>A | R345Q |
| 85. | NM_000084.5:c.1087G>A | V363M |
| 86. | NM_000084.5:c.1121A>G | N374S |
| 87. | NM_000084.5:c.1144A>G | S382G |
| 88. | NM_000084.5:c.1172A>C | D391A |
| 89. | NM_000084.5:c.1184T>G | L395R |
| 90. | NM_000084.5:c.1186G>C | D396H |
| 91. | NM_000084.5:c.1198C>T | L400F |
| 92. | NM_000084.5:c.1212G>C | E404D |
| 93. | NM_000084.5:c.1210G>A | E404K |
| 94. | NM_000084.5:c.1216C>T | R406C |
| 95. | NM_000084.5:c.1217G>A | R406H |
| 96. | NM_000084.5:c.1256C>T | P419L |
| 97. | NM_000084.5:c.1264G>A | V422M |
| 98. | NM_000084.5:c.1276A>G | S426G |
| 99. | NM_000084.5:c.1285T>C | W429R |
| 100. | NM_000084.5:c.1327A>G | I443V |
| 101. | CM1314272 NM_000084.5:c.1384G>A | G462S |
| 102. | CM044218 NM_000084.5:c.1385G>A | G462S |
| 103. | CM052172 NM_000084.5:c.1385G>T | G462D |
| 104. | CM125772 NM_000084.5:c.1396G>C | G466R |
| 105. | CM1314274 NM_000084.5:c.1397G>A | G466D |
| 106. | NM_000084.5:c.1397G>T | G466V |
| 107. | CM139679 NM_000084.5:c.1403T>C | L468P |
| 108. | CM097074 NM_000084.5:c.1406T>C | L469P |
| 109. | CM157780 NM_000084.5:c.1408G>A | G470R |
| 110. | NM_000084.5:c.1418T>C | M473T |
| 111. | NM_000084.5:c.1425G>T | Q475H |
| 112. | NM_000084.5:c.1433A>G | Y478C |
| 113. | NM_000084.5:c.1464C>G | S488R |
| 114. | NM_000084.5:c.1475A>G | Q492R |
| 115. | NM_000084.5:c.1483G>T | D495Y |
| 116. | CM139682 NM_000084.5:c.1505A>G | Y502C |
| 117. | NM_000084.5:c.1510A>G | M504V |
| 118. | CM097570 NM_000084.5:c.1511T>A | M504K |
| 119. | CM109654 NM_000084.5:c.1514T>G | V505G |
| 120. | CM139683 NM_000084.5:c.1516G>A | G506R |
| 121. | CM960311 NM_000084.5:c.1517G>A | G506E |
| 122. | CM970318 NM_000084.5:c.1534G>C | G512R |
| 123. | CM095946 NM_000084.5:c.1535G>A | G512D |
| 124. | NM_000084.5:c.1535G>T | G512V |
| 125. | CM044219 NM_000084.5:c.1537G>A | G513R |
| 126. | CM973255 NM_000084.5:c.1538G>A | G513E |
| 127. | CM139684 NM_000084.5:c.1547G>A | R516Q |
| 128. | CM973256 NM_000084.5:c.1546C>T | R516W |
| 129. | CM157781 NM_000084.5:c.1552A>G | T518A |
| 130. | CM095947 NM_000084.5:c.1556T>A | V519D |
| 131. | CM960312 NM_000084.5:c.1558T>C | S520P |
| 132. | CM146435 NM_000084.5:c.1561C>T | L521F |
| 133. | NM_000084.5:c.1564G>T | V522F |
| 134. | CM010188 NM_000084.5:c.1571T>A | I524K |
| 135. | CM1617183 NM_000084.5:c.1579G>C | E527Q |
| 136. | CM970319 NM_000084.5:c.1581A>T | E527D |
| 137. | NM_000084.5:c.1586C>T | T529I |
| 138. | CM157782 NM_000084.5:c.1588G>A | G530S |
| 139. | CM1718625 NM_000084.5:c.1589G>T | G530V |
| 140. | NM_000084.5:c.1600T>A | Y534N |
| 141. | NM_000084.5:c.1606G>A | V536M |
| 142. | CM1613493 NM_000084.5:c.1618G>C | A540P |
| 143. | NM_000084.5:c.1618G>A | A540T |
| 144. | CM044220 NM_000084.5:c.1634G>A | S545N |
| 145. | CM1718627 NM_000084.5:c.1633A>C | S545R |
| 146. | CM044221 NM_000084.5:c.1636A>G | K546E |
| 147. | NM_000084.5:c.1637A>G | K546R |
| 148. | CM095948 NM_000084.5:c.1639T>C | W547R |
| 149. | CM071609 NM_000084.5:c.1639T>G | W547G |
| 150. | NM_000084.5:c.1641G>T | W547C |
| 151. | NM_000084.5:c.1642G>A | V548M |
| 152. | NM_000084.5:c.1663G>A | E555K |
| 153. | NM_000084.5:c.1688G>A | R563H |
| 154. | NM_000084.5:c.1707T>G | F569L |
| 155. | NM_000084.5:c.1732C>T | H578Y |
| 156. | NM_000084.5:c.1766G>A | R589Q |
| 157. | NM_000084.5:c.1774G>A | D592N |
| 158. | CM004240 NM_000084.5:c.1851C>A | Y617I |
| 159. | CM095949 NM_000084.5:c.1862C>T | P621L |
| 160. | NM_000084.5:c.1877G>A | R626Q |
| 161. | NM_000084.5:c.1906C>T | L636F |
| 162. | NM_000084.5:c.1921A>G | I641V |
| 163. | NM_000084.5:c.1943G>A | R648Q |
| 164. | NM_000084.5:c.1958G>C | G653A |
| 165. | CM044222 NM_000084.5:c.1970C>G | T657S |
| 166. | NM_000084.5:c.1978A>G | I660V |
| 167. | NM_000084.5:c.1988C>T | T663M |
| 168. | NM_000084.5:c.1987A>G | T663A |
| 169. | NM_000084.5:c.1991A>C | E664A |
| 170. | NM_000084.5:c.2011C>T | P671S |
| 171. | NM_000084.5:c.2039G>A | R680Q |
| 172. | NM_000084.5:c.2051A>G | D684G |
| 173. | NM_000084.5:c.2083C>T | P695S |
| 174. | NM_000084.5:c.2095G>A | V699I |
| 175. | CM139686 NM_000084.5:c.2108T>C | F703S |
| 176. | NM_000084.5:c.2111G>A | R704Q |
| 177. | CM139687 NM_000084.5:c.2117T>C | L706P |
| 178. | NM_000084.5:c.2131T>C | C711R |
| 179. | CM139688 NM_000084.5:c.2133C>G | C711W |
| 180. | NM_000084.5:c.2132G>C | C711S |
| 181. | CM066010 NM_000084.5:c.2173A>G | K725E |

Table S2. Deleteriousness prediction of CLCN5 mutations using the PredictSNP server.

| **Accession Number** | **Amino acid change** | **PredictSNP** | **MAPP** | **PhDUSNP** | **PolyPhenU1** | **PolyPhenU2** | **SIFT** | **SNAP** | **PANTHER** |
| --- | --- | --- | --- | --- | --- | --- | --- | --- | --- |
| **CM983857** | **W22G** | **D** | **D** | **D** | **D** | **D** | **D** | **D** | **U** |
| **CM187918** | **R30W** | **D** | **N** | **D** | **D** | **D** | **D** | **D** | **U** |
| **CM1617184** | **G57R** | **D** | **N** | **D** | **D** | **D** | **D** | **N** | **U** |
| **CM970315** | **G57V** | **N** | **N** | **D** | **D** | **N** | **D** | **N** | **U** |
| **CM097568** | **W58C** | **D** | **D** | **D** | **D** | **D** | **D** | **D** | **D** |
| **CM095944** | **W58L** | **D** | **D** | **D** | **D** | **N** | **D** | **N** | **N** |
| **CM1314273** | **G65R** | **D** | **D** | **D** | **D** | **D** | **D** | **D** | **D** |
| **NM_000084.5:c.238C>A** | **H80N** | **N** | **N** | **N** | **N** | **N** | **N** | **N** | **N** |
| **CM066011** | **G88D** | **D** | **D** | **D** | **D** | **D** | **D** | **D** | **U** |
| **CM139666** | **G88V** | **D** | **D** | **D** | **D** | **D** | **D** | **D** | **U** |
| **NM_000084.5:c.268T>C** | **C90R** | **D** | **D** | **D** | **D** | **D** | **D** | **D** | **U** |
| **CM139667** | **C90W** | **D** | **D** | **D** | **D** | **D** | **D** | **D** | **U** |
| **CM1414749** | **H100R** | **N** | **N** | **N** | **N** | **N** | **N** | **D** | **U** |
| **CM082570** | **C101Y** | **D** | **N** | **D** | **D** | **D** | **D** | **D** | **U** |
| **CM139669** | **W103R** | **D** | **U** | **D** | **D** | **D** | **D** | **D** | **U** |
| **NM_000084.5:c.344A>G** | **K115R** | **N** | **U** | **N** | **N** | **N** | **N** | **N** | **U** |
| **NM_000084.5:c.373C>G** | **L125V** | **N** | **N** | **N** | **N** | **N** | **N** | **N** | **N** |
| **CM1414345** | **D130G** | **N** | **U** | **N** | **N** | **N** | **N** | **N** | **N** |
| **NM_000084.5:c.426G>A** | **M142I** | **N** | **N** | **D** | **N** | **N** | **N** | **N** | **N** |
| **NM_000084.5:c.472G>C** | **V158L** | **N** | **N** | **D** | **N** | **D** | **D** | **N** | **N** |
| **CM139671** | **I176N** | **D** | **D** | **D** | **D** | **D** | **D** | **N** | **U** |
| **CM097071** | **G179D** | **D** | **D** | **D** | **D** | **D** | **D** | **D** | **U** |
| **NM_000084.5:c.586A>G** | **I196V** | **N** | **N** | **N** | **N** | **N** | **N** | **N** | **N** |
| **NM_000084.5:c.599T>G** | **L200R** | **D** | **D** | **D** | **D** | **D** | **D** | **D** | **U** |
| **CM097072** | **S203L** | **D** | **D** | **N** | **D** | **D** | **D** | **D** | **D** |
| **CM139673** | **E211Q** | **D** | **N** | **D** | **D** | **D** | **D** | **D** | **U** |
| **CM139674** | **G212A** | **D** | **D** | **D** | **N** | **D** | **D** | **D** | **U** |
| **CM097073** | **G212S** | **D** | **D** | **D** | **N** | **D** | **D** | **D** | **U** |
| **CM139675** | **P213L** | **D** | **D** | **D** | **D** | **D** | **D** | **D** | **U** |
| **CM071612** | **C219R** | **D** | **D** | **D** | **D** | **D** | **D** | **D** | **D** |
| **NM_000084.5:c.661T>C** | **C221R** | **D** | **D** | **D** | **D** | **D** | **D** | **D** | **U** |
| **CM077238** | **L225P** | **D** | **D** | **D** | **D** | **D** | **D** | **N** | **D** |
| **CM157776** | **K231I** | **D** | **D** | **N** | **D** | **D** | **D** | **D** | **U** |
| **CM157777** | **R239C** | **D** | **D** | **D** | **D** | **D** | **D** | **D** | **D** |
| **NM_000084.5:c.716G>A** | **R239H** | **D** | **N** | **N** | **D** | **D** | **D** | **D** | **N** |
| **NM_000084.5:c.715C>T** | **R239P** | **D** | **D** | **D** | **D** | **D** | **D** | **D** | **D** |
| **CM960309** | **S244L** | **D** | **D** | **D** | **D** | **D** | **D** | **D** | **U** |
| **NM_000084.5:c.749G>A** | **G250D** | **D** | **D** | **D** | **D** | **D** | **D** | **D** | **U** |
| **CM157778** | **G250R** | **D** | **D** | **D** | **D** | **D** | **D** | **D** | **U** |
| **NM_000084.5:c.751G>A** | **V251I** | **N** | **N** | **N** | **N** | **D** | **D** | **N** | **U** |
| **NM_000084.5:c.766G>C** | **G256R** | **D** | **D** | **D** | **D** | **D** | **D** | **D** | **U** |
| **NM_000084.5:c.779G>A** | **G260D** | **D** | **D** | **D** | **D** | **D** | **D** | **D** | **U** |
| **CM062520** | **G260V** | **D** | **D** | **D** | **D** | **D** | **D** | **D** | **U** |
| **CM095945** | **G261E** | **D** | **D** | **D** | **D** | **D** | **D** | **D** | **U** |
| **CM104899** | **G261R** | **D** | **D** | **D** | **D** | **D** | **D** | **D** | **U** |
| **CM166359** | **L263F** | **D** | **D** | **D** | **D** | **D** | **D** | **D** | **U** |
| **CM166360** | **S265R** | **D** | **D** | **D** | **D** | **D** | **D** | **D** | **U** |
| **CM139676** | **L266V** | **D** | **D** | **D** | **N** | **D** | **D** | **N** | **U** |
| **CM153737** | **E267A** | **D** | **D** | **D** | **D** | **D** | **D** | **D** | **U** |
| **CM157779** | **E267D** | **D** | **D** | **D** | **D** | **D** | **D** | **D** | **U** |
| **CM185956** | **E267V** | **D** | **D** | **D** | **D** | **D** | **D** | **D** | **U** |
| **CM1516353** | **E268D** | **D** | **N** | **D** | **D** | **D** | **D** | **N** | **U** |
| **CM044217** | **S270G** | **D** | **N** | **D** | **D** | **D** | **D** | **N** | **N** |
| **CM980369** | **S270R** | **D** | **D** | **D** | **D** | **D** | **D** | **D** | **N** |
| **CM066756** | **Y272C** | **D** | **N** | **D** | **D** | **D** | **D** | **D** | **U** |
| **CM139677** | **Y272N** | **D** | **D** | **D** | **D** | **D** | **D** | **N** | **U** |
| **CM071613** | **F273L** | **D** | **D** | **D** | **N** | **D** | **D** | **N** | **U** |
| **NM_000084.5:c.829A>C** | **T277P** | **D** | **D** | **D** | **D** | **D** | **D** | **D** | **D** |
| **CM980370** | **L278F** | **D** | **N** | **D** | **N** | **D** | **D** | **N** | **N** |
| **CM166356** | **L278S** | **D** | **N** | **D** | **D** | **D** | **D** | **D** | **N** |
| **CM122880** | **L278W** | **D** | **N** | **D** | **D** | **D** | **D** | **D** | **D** |
| **CM970316** | **R280P** | **D** | **D** | **D** | **D** | **D** | **D** | **D** | **D** |
| **NM_000084.5:c.851C>T** | **A284V** | **N** | **N** | **D** | **N** | **N** | **D** | **N** | **N** |
| **NM_000084.5:c.877C>T** | **R293C** | **D** | **D** | **D** | **D** | **D** | **D** | **D** | **U** |
| **NM_000084.5:c.898A>G** | **N300D** | **N** | **N** | **N** | **N** | **N** | **D** | **N** | **N** |
| **NM_000084.5:c.904C>T** | **R302C** | **D** | **N** | **D** | **D** | **D** | **D** | **N** | **D** |
| **NM_000084.5:c.956T>G** | **L319R** | **D** | **D** | **D** | **D** | **D** | **D** | **D** | **U** |
| **CM052170** | **L324R** | **D** | **D** | **D** | **D** | **D** | **D** | **D** | **D** |
| **CM122879** | **G329D** | **D** | **D** | **D** | **D** | **D** | **D** | **D** | **U** |
| **CM077523** | **G333R** | **D** | **D** | **D** | **D** | **D** | **D** | **D** | **D** |
| **NM_000084.5:c.1012C>T** | **R338C** | **D** | **D** | **D** | **D** | **D** | **D** | **D** | **D** |
| **NM_000084.5:c.1013G>A** | **R338H** | **N** | **N** | **N** | **D** | **N** | **N** | **N** | **N** |
| **CM066755** | **N340K** | **D** | **D** | **D** | **D** | **N** | **D** | **D** | **U** |
| **NM_000084.5:c.1034G>T** | **R345L** | **N** | **N** | **D** | **D** | **N** | **D** | **N** | **N** |
| **NM_000084.5:c.1034G>A** | **R345Q** | **N** | **N** | **N** | **D** | **N** | **D** | **N** | **N** |
| **CM165162** | **R345W** | **D** | **N** | **D** | **D** | **D** | **D** | **N** | **D** |
| **NM_000084.5:c.1121A>G** | **N374S** | **N** | **N** | **N** | **N** | **N** | **D** | **N** | **N** |
| **NM_000084.5:c.1172A>C** | **D391A** | **N** | **D** | **D** | **N** | **N** | **D** | **N** | **N** |
| **NM_000084.5:c.1184T>G** | **L395R** | **N** | **N** | **N** | **N** | **D** | **N** | **N** | **D** |
| **NM_000084.5:c.1186G>C** | **D396H** | **D** | **N** | **N** | **D** | **D** | **D** | **N** | **N** |
| **NM_000084.5:c.1216C>T** | **R406C** | **N** | **U** | **D** | **D** | **N** | **N** | **N** | **N** |
| **NM_000084.5:c.1217G>A** | **R406H** | **N** | **U** | **N** | **N** | **N** | **N** | **N** | **N** |
| **NM_000084.5:c.1256C>T** | **P419L** | **N** | **U** | **D** | **N** | **N** | **D** | **N** | **N** |
| **NM_000084.5:c.1264G>A** | **V422M** | **N** | **N** | **N** | **N** | **D** | **N** | **N** | **N** |
| **NM_000084.5:c.1327A>G** | **I443V** | **N** | **N** | **N** | **N** | **N** | **N** | **N** | **N** |
| **CM052172** | **G462D** | **D** | **D** | **D** | **D** | **D** | **D** | **D** | **U** |
| **CM044218** | **G462S** | **D** | **D** | **D** | **N** | **D** | **D** | **D** | **U** |
| **CM1314274** | **G466D** | **D** | **D** | **D** | **D** | **D** | **D** | **D** | **U** |
| **CM125772** | **G466R** | **D** | **D** | **D** | **D** | **D** | **D** | **D** | **U** |
| **NM_000084.5:c.1397G>T** | **G466V** | **D** | **D** | **D** | **D** | **D** | **D** | **D** | **U** |
| **CM139679** | **L468P** | **D** | **D** | **D** | **D** | **D** | **D** | **D** | **U** |
| **CM097074** | **L469P** | **D** | **D** | **D** | **D** | **D** | **D** | **D** | **D** |
| **CM157780** | **G470R** | **D** | **D** | **D** | **D** | **D** | **D** | **D** | **U** |
| **NM_000084.5:c.1425G>T** | **Q475H** | **N** | **N** | **N** | **N** | **N** | **D** | **N** | **U** |
| **NM_000084.5:c.1433A>G** | **Y478C** | **D** | **N** | **D** | **D** | **N** | **D** | **N** | **D** |
| **NM_000084.5:c.1464C>G** | **S488R** | **D** | **D** | **D** | **N** | **N** | **N** | **D** | **N** |
| **NM_000084.5:c.1475A>G** | **Q492R** | **N** | **D** | **N** | **N** | **N** | **N** | **N** | **U** |
| **CM139682** | **Y502C** | **D** | **D** | **D** | **D** | **D** | **D** | **D** | **U** |
| **NM_000084.5:c.1510A>G** | **M504V** | **N** | **D** | **D** | **N** | **N** | **D** | **N** | **U** |
| **CM097570** | **M504K** | **D** | **D** | **D** | **D** | **D** | **D** | **D** | **U** |
| **CM109654** | **V505G** | **D** | **D** | **D** | **D** | **D** | **D** | **D** | **U** |
| **CM960311** | **G506E** | **D** | **D** | **D** | **D** | **D** | **D** | **D** | **U** |
| **CM139683** | **G506R** | **D** | **D** | **D** | **D** | **D** | **D** | **D** | **U** |
| **CM095946** | **G512D** | **D** | **D** | **D** | **D** | **N** | **D** | **D** | **U** |
| **CM970318** | **G512R** | **D** | **D** | **D** | **D** | **D** | **D** | **D** | **U** |
| **NM_000084.5:c.1535G>T** | **G512V** | **D** | **D** | **D** | **D** | **D** | **D** | **D** | **U** |
| **CM973255** | **G513E** | **D** | **D** | **D** | **D** | **D** | **D** | **D** | **U** |
| **CM044219** | **G513R** | **D** | **D** | **D** | **D** | **D** | **D** | **D** | **U** |
| **CM139684** | **R516Q** | **D** | **N** | **D** | **D** | **D** | **D** | **D** | **U** |
| **CM973256** | **R516W** | **D** | **D** | **D** | **D** | **D** | **D** | **D** | **U** |
| **CM157781** | **T518A** | **D** | **D** | **D** | **D** | **D** | **D** | **D** | **U** |
| **CM095947** | **V519D** | **D** | **D** | **D** | **D** | **D** | **D** | **D** | **U** |
| **CM960312** | **S520P** | **D** | **D** | **D** | **D** | **D** | **D** | **D** | **U** |
| **CM146435** | **L521F** | **D** | **N** | **N** | **D** | **D** | **D** | **D** | **U** |
| **NM_000084.5:c.1564G>T** | **V522F** | **D** | **D** | **D** | **D** | **D** | **D** | **D** | **U** |
| **CM010188** | **I524K** | **D** | **D** | **D** | **D** | **D** | **D** | **D** | **U** |
| **CM1617183** | **E527D** | **D** | **D** | **D** | **D** | **D** | **D** | **D** | **U** |
| **CM970319** | **E527Q** | **D** | **D** | **D** | **D** | **D** | **D** | **D** | **U** |
| **NM_000084.5:c.1586C>T** | **T529I** | **D** | **D** | **D** | **D** | **D** | **D** | **D** | **U** |
| **CM157782** | **G530S** | **D** | **N** | **D** | **D** | **D** | **D** | **N** | **U** |
| **CM1718625** | **G530V** | **D** | **D** | **D** | **D** | **D** | **D** | **D** | **U** |
| **NM_000084.5:c.1600T>A** | **Y534N** | **D** | **N** | **D** | **D** | **D** | **D** | **N** | **U** |
| **CM1613493** | **A540P** | **D** | **D** | **D** | **D** | **N** | **D** | **D** | **U** |
| **NM_000084.5:c.1618G>A** | **A540T** | **N** | **D** | **N** | **N** | **N** | **N** | **N** | **U** |
| **CM044220** | **S545N** | **D** | **D** | **D** | **N** | **D** | **D** | **D** | **U** |
| **CM1718627** | **S545R** | **D** | **D** | **D** | **D** | **D** | **D** | **D** | **U** |
| **CM044221** | **K546E** | **D** | **D** | **D** | **D** | **D** | **D** | **D** | **U** |
| **NM_000084.5:c.1637A>G** | **K546R** | **D** | **N** | **D** | **D** | **D** | **D** | **N** | **U** |
| **NM_000084.5:c.1641G>T** | **W547C** | **D** | **N** | **D** | **D** | **D** | **D** | **D** | **U** |
| **CM071609** | **W547G** | **D** | **N** | **D** | **D** | **N** | **D** | **D** | **U** |
| **CM095948** | **W547R** | **D** | **D** | **D** | **D** | **D** | **D** | **D** | **U** |
| **NM_000084.5:c.1642G>A** | **V548M** | **D** | **D** | **D** | **N** | **D** | **D** | **N** | **U** |
| **NM_000084.5:c.1707T>G** | **F569L** | **N** | **N** | **D** | **N** | **N** | **D** | **N** | **U** |
| **NM_000084.5:c.1774G>A** | **D592N** | **N** | **N** | **N** | **N** | **N** | **N** | **N** | **U** |
| **CM004240** | **Y617I** | **N** | **N** | **N** | **N** | **N** | **D** | **D** | **D** |
| **CM095949** | **P621L** | **D** | **D** | **D** | **D** | **D** | **D** | **D** | **U** |
| **NM_000084.5:c.1877G>A** | **R626Q** | **N** | **N** | **N** | **N** | **N** | **N** | **N** | **N** |
| **NM_000084.5:c.1906C>T** | **L636F** | **N** | **N** | **N** | **D** | **D** | **N** | **N** | **N** |
| **NM_000084.5:c.1958G>C** | **G653A** | **N** | **N** | **N** | **N** | **N** | **D** | **N** | **N** |
| **CM044222** | **T657S** | **N** | **N** | **N** | **N** | **N** | **N** | **N** | **N** |
| **NM_000084.5:c.1978A>G** | **I660V** | **N** | **N** | **N** | **N** | **N** | **N** | **N** | **N** |
| **NM_000084.5:c.1987A>G** | **T663A** | **N** | **N** | **N** | **N** | **N** | **N** | **N** | **N** |
| **NM_000084.5:c.1988C>T** | **T663M** | **N** | **N** | **N** | **D** | **D** | **D** | **N** | **D** |
| **NM_000084.5:c.1991A>C** | **E664A** | **N** | **N** | **N** | **N** | **N** | **N** | **N** | **N** |
| **NM_000084.5:c.2051A>G** | **D684G** | **D** | **D** | **D** | **D** | **D** | **D** | **D** | **U** |
| **NM_000084.5:c.2083C>T** | **P695S** | **N** | **N** | **N** | **N** | **D** | **N** | **N** | **N** |
| **NM_000084.5:c.2095G>A** | **V699I** | **N** | **N** | **N** | **N** | **D** | **D** | **N** | **U** |
| **CM139686** | **F703S** | **D** | **D** | **D** | **D** | **D** | **D** | **D** | **U** |
| **NM_000084.5:c.2111G>A** | **R704Q** | **N** | **N** | **N** | **N** | **D** | **D** | **N** | **N** |
| **CM139687** | **L706P** | **D** | **D** | **D** | **D** | **D** | **D** | **D** | **U** |
| **NM_000084.5:c.2132G>C** | **C711S** | **N** | **D** | **N** | **N** | **N** | **D** | **N** | **N** |
| **CM139688** | **C711W** | **D** | **D** | **D** | **D** | **D** | **D** | **D** | **D** |
| **CM066010** | **K725E** | **D** | **D** | **D** | **D** | **D** | **D** | **N** | **U** |

D—Deleterious, N—Neutral, U—Unknown

Table S3. Predict the pathogenicity of CLCN5(NM_000084.5) mutations using the MAGPIE website.

| Accession Number | Accession number | Chr | Start | End | MAGPIE prediction |
| --- | --- | --- | --- | --- | --- |
| NM_000084.5:c.64T>G | W22G | X | 50069989 | 50069989 | 0.856377204 |
| NM_000084.5:c.88C>T | R30W | X | 50070013 | 50070013 | 0.462045272 |
| NM_000084.5:c.169G>C | G57R | X | 50072552 | 50072552 | 0.969879208 |
| NM_000084.5:c.169G>T | G57V | X | 50072552 | 50072552 | 0.928794141 |
| NM_000084.5:c.174G>C | W58C | X | 50072557 | 50072557 | 0.790901275 |
| NM_000084.5:c.173G>T | W58L | X | 50072556 | 50072556 | 0.914268768 |
| NM_000084.5:c.193G>A | G65R | X | 50072576 | 50072576 | 0.964034124 |
| NM_000084.5:c.238C>A | H80N | X | 50075827 | 50075827 | 0.202866611 |
| NM_000084.5:c.263G>A | G88D | X | 50075852 | 50075852 | 0.969980183 |
| NM_000084.5:c.263G>T | G88V | X | 50075852 | 50075852 | 0.963426798 |
| NM_000084.5:c.268T>C | C90R | X | 50075857 | 50075857 | 0.924236 |
| NM_000084.5:c.270C>G | C90W | X | 50075859 | 50075859 | 0.803472383 |
| NM_000084.5:c.299A>G | H100R | X | 50075888 | 50075888 | 0.834526116 |
| NM_000084.5:c.302G>A | C101Y | X | 50075891 | 50075891 | 0.970804814 |
| NM_000084.5:c.307T>C | W103R | X | 50075896 | 50075896 | 0.945800359 |
| NM_000084.5:c.344A>G | K115R | X | 50075933 | 50075933 | 0.001549378 |
| NM_000084.5:c.373C>G | L125V | X | 50075962 | 50075962 | 0.467205324 |
| NM_000084.5:c.389A>G | D130G | X | 50075978 | 50075978 | 0.736073354 |
| NM_000084.5:c.426G>A | M142I | X | 50080626 | 50080626 | 0.000131292 |
| NM_000084.5:c.472G>C | V158L | X | 50080672 | 50080672 | 0.492989573 |
| NM_000084.5:c.527T>A | I176N | X | 50081651 | 50081651 | 0.929168739 |
| NM_000084.5:c.536G>A | G179D | X | 50081660 | 50081660 | 0.926178084 |
| NM_000084.5:c.586A>G | I196V | X | 50081710 | 50081710 | 0.00152906 |
| NM_000084.5:c.599T>G | L200R | X | 50081723 | 50081723 | 0.936556742 |
| NM_000084.5:c.608C>T | S203L | X | 50081732 | 50081732 | 0.864530733 |
| NM_000084.5:c.631G>C | E211Q | X | 50081755 | 50081755 | 0.63951287 |
| NM_000084.5:c.634G>A | G212S | X | 50081758 | 50081758 | 0.930216343 |
| NM_000084.5:c.635G>C | G212A | X | 50081759 | 50081759 | 0.885059007 |
| NM_000084.5:c.638C>T | P213L | X | 50081762 | 50081762 | 0.849510878 |
| NM_000084.5:c.655T>C | C219R | X | 50081779 | 50081779 | 0.984493565 |
| NM_000084.5:c.661T>C | C221R | X | 50081785 | 50081785 | 0.974246783 |
| NM_000084.5:c.674T>C | L225P | X | 50081798 | 50081798 | 0.877896768 |
| NM_000084.5:c.692A>T | K231I | X | 50081816 | 50081816 | 0.793167034 |
| NM_000084.5:c.716G>C | R239P | X | 50081840 | 50081840 | 0.851964255 |
| NM_000084.5:c.716G>A | R239H | X | 50081840 | 50081840 | 0.034730226 |
| NM_000084.5:c.715C>T | R239C | X | 50081839 | 50081839 | 0.443048992 |
| NM_000084.5:c.731C>T | S244L | X | 50085987 | 50085987 | 0.908437252 |
| NM_000084.5:c.748G>C | G250R | X | 50086004 | 50086004 | 0.944234576 |
| NM_000084.5:c.749G>A | G250D | X | 50086005 | 50086005 | 0.873865 |
| NM_000084.5:c.751G>A | V251I | X | 50086007 | 50086007 | 0.803471458 |
| NM_000084.5:c.766G>C | G256R | X | 50086022 | 50086022 | 0.844520657 |
| NM_000084.5:c.779G>A | G260D | X | 50086035 | 50086035 | 0.450356 |
| NM_000084.5:c.779G>T | G260V | X | 50086035 | 50086035 | 0.917517367 |
| NM_000084.5:c.781G>A | G261R | X | 50086037 | 50086037 | 0.981211145 |
| NM_000084.5:c.782G>A | G261E | X | 50086038 | 50086038 | 0.928505472 |
| NM_000084.5:c.789A>T | L263F | X | 50086045 | 50086045 | 0.069032277 |
| NM_000084.5:c.793A>C | S265R | X | 50086049 | 50086049 | 0.698153066 |
| NM_000084.5:c.796C>G | L266V | X | 50086052 | 50086052 | 0.075038919 |
| NM_000084.5:c.799G>A | E267K | X | 50086055 | 50086055 | 0.839399359 |
| NM_000084.5:c.801A>C | E267D | X | 50086057 | 50086057 | 0.182378383 |
| NM_000084.5:c.800A>C | E267A | X | 50086056 | 50086056 | 0.417855446 |
| NM_000084.5:c.800A>T | E267V | X | 50086056 | 50086056 | 0.444598301 |
| NM_000084.5:c.804G>C | E268D | X | 50086060 | 50086060 | 0.990566138 |
| NM_000084.5:c.810C>G | S270R | X | 50086333 | 50086333 | 0.55322776 |
| NM_000084.5:c.808A>C | S270R | X | 50086331 | 50086331 | 0.793181858 |
| NM_000084.5:c.808A>G | S270G | X | 50086331 | 50086331 | 0.842258667 |
| NM_000084.5:c.814T>A | Y272N | X | 50086337 | 50086337 | 0.912301622 |
| NM_000084.5:c.815A>G | Y272C | X | 50086338 | 50086338 | 0.894669998 |
| NM_000084.5:c.817T>C | F273L | X | 50086340 | 50086340 | 0.70416584 |
| NM_000084.5:c.829A>C | T277P | X | 50086352 | 50086352 | 0.948989 |
| NM_000084.5:c.834G>C | L278F | X | 50086357 | 50086357 | 0.252767608 |
| NM_000084.5:c.833T>C | L278S | X | 50086356 | 50086356 | 0.892990704 |
| NM_000084.5:c.833T>G | L278W | X | 50086356 | 50086356 | 0.914036624 |
| NM_000084.5:c.839G>C | R280P | X | 50086362 | 50086362 | 0.956300175 |
| NM_000084.5:c.851C>T | A284V | X | 50086374 | 50086374 | 0.786638028 |
| NM_000084.5:c.877C>T | R293C | X | 50086400 | 50086400 | 0.596450094 |
| NM_000084.5:c.898A>G | N300D | X | 50086421 | 50086421 | 0.56952974 |
| NM_000084.5:c.904C>T | R302C | X | 50086427 | 50086427 | 0.604120371 |
| NM_000084.5:c.956T>G | L319R | X | 50086479 | 50086479 | 0.015817 |
| NM_000084.5:c.971T>G | L324R | X | 50086494 | 50086494 | 0.729787845 |
| NM_000084.5:c.986G>A | G329D | X | 50086509 | 50086509 | 0.896013922 |
| NM_000084.5:c.997G>A | G333R | X | 50086520 | 50086520 | 0.95869786 |
| NM_000084.5:c.1013G>A | R338H | X | 50086536 | 50086536 | 0.043604485 |
| NM_000084.5:c.1012C>T | R338C | X | 50086535 | 50086535 | 0.3447286 |
| NM_000084.5:c.1020C>G | N340K | X | 50086543 | 50086543 | 0.211599696 |
| NM_000084.5:c.1033C>T | R345W | X | 50086556 | 50086556 | 0.544796981 |
| NM_000084.5:c.1034G>T | R345L | X | 50086557 | 50086557 | 0.792434407 |
| NM_000084.5:c.1034G>A | R345Q | X | 50086557 | 50086557 | 0.054807775 |
| NM_000084.5:c.1121A>G | N374S | X | 50086644 | 50086644 | 0.088285198 |
| NM_000084.5:c.1172A>C | D391A | X | 50086695 | 50086695 | 0.728014018 |
| NM_000084.5:c.1184T>G | L395R | X | 50086707 | 50086707 | 0.874461052 |
| NM_000084.5:c.1186G>C | D396H | X | 50086709 | 50086709 | 0.782657338 |
| NM_000084.5:c.1216C>T | R406C | X | 50086739 | 50086739 | 0.053931875 |
| NM_000084.5:c.1217G>A | R406H | X | 50086740 | 50086740 | 0.070155028 |
| NM_000084.5:c.1256C>T | P419L | X | 50086779 | 50086779 | 0.361196996 |
| NM_000084.5:c.1264G>A | V422M | X | 50086787 | 50086787 | 0.003795934 |
| NM_000084.5:c.1327A>G | I443V | X | 50086850 | 50086850 | 0.009736121 |
| NM_000084.5:c.1384G>A | G462S | X | 50088734 | 50088734 | 0.946993776 |
| NM_000084.5:c.1385G>A | G462S | X | 50088735 | 50088735 | 0.957519972 |
| NM_000084.5:c.1385G>T | G462D | X | 50088735 | 50088735 | 0.944924473 |
| NM_000084.5:c.1396G>C | G466R | X | 50088746 | 50088746 | 0.970890541 |
| NM_000084.5:c.1397G>A | G466D | X | 50088747 | 50088747 | 0.957406986 |
| NM_000084.5:c.1397G>T | G466V | X | 50088747 | 50088747 | 0.971355026 |
| NM_000084.5:c.1403T>C | L468P | X | 50088753 | 50088753 | 0.961475526 |
| NM_000084.5:c.1406T>C | L469P | X | 50088756 | 50088756 | 0.956159599 |
| NM_000084.5:c.1408G>A | G470R | X | 50088758 | 50088758 | 0.939930412 |
| NM_000084.5:c.1425G>T | Q475H | X | 50088775 | 50088775 | 0.002089095 |
| NM_000084.5:c.1433A>G | Y478C | X | 50088783 | 50088783 | 0.273200664 |
| NM_000084.5:c.1464C>G | S488R | X | 50088814 | 50088814 | 0.218008289 |
| NM_000084.5:c.1475A>G | Q492R | X | 50088825 | 50088825 | 0.065177165 |
| NM_000084.5:c.1505A>G | Y502C | X | 50088855 | 50088855 | 0.961192478 |
| NM_000084.5:c.1510A>G | M504V | X | 50088860 | 50088860 | 0.011787 |
| NM_000084.5:c.1511T>A | M504K | X | 50088861 | 50088861 | 0.946511448 |
| NM_000084.5:c.1514T>G | V505G | X | 50088864 | 50088864 | 0.829232845 |
| NM_000084.5:c.1516G>A | G506R | X | 50088866 | 50088866 | 0.972051479 |
| NM_000084.5:c.1517G>A | G506E | X | 50088867 | 50088867 | 0.962833128 |
| NM_000084.5:c.1534G>C | G512R | X | 50088884 | 50088884 | 0.932351134 |
| NM_000084.5:c.1535G>A | G512D | X | 50090116 | 50090116 | 0.944073129 |
| NM_000084.5:c.1535G>T | G512V | X | 50090116 | 50090116 | 0.963235043 |
| NM_000084.5:c.1537G>A | G513R | X | 50090118 | 50090118 | 0.974087893 |
| NM_000084.5:c.1538G>A | G513E | X | 50090119 | 50090119 | 0.956194787 |
| NM_000084.5:c.1547G>A | R516Q | X | 50090128 | 50090128 | 0.897595248 |
| NM_000084.5:c.1546C>T | R516W | X | 50090127 | 50090127 | 0.945780598 |
| NM_000084.5:c.1552A>G | T518A | X | 50090133 | 50090133 | 0.842993313 |
| NM_000084.5:c.1556T>A | V519D | X | 50090137 | 50090137 | 0.971812426 |
| NM_000084.5:c.1558T>C | S520P | X | 50090139 | 50090139 | 0.97049109 |
| NM_000084.5:c.1561C>T | L521F | X | 50090142 | 50090142 | 0.814767525 |
| NM_000084.5:c.1564G>T | V522F | X | 50090145 | 50090145 | 0.035561 |
| NM_000084.5:c.1571T>A | I524K | X | 50090152 | 50090152 | 0.969723547 |
| NM_000084.5:c.1579G>C | E527Q | X | 50090160 | 50090160 | 0.654035402 |
| NM_000084.5:c.1581A>T | E527D | X | 50090162 | 50090162 | 0.07192312 |
| NM_000084.5:c.1586C>T | T529I | X | 50090167 | 50090167 | 0.873227752 |
| NM_000084.5:c.1588G>A | G530S | X | 50090169 | 50090169 | 0.958442256 |
| NM_000084.5:c.1589G>T | G530V | X | 50090170 | 50090170 | 0.968923475 |
| NM_000084.5:c.1600T>A | Y534N | X | 50090181 | 50090181 | 0.815937837 |
| NM_000084.5:c.1618G>C | A540P | X | 50090199 | 50090199 | 0.981884319 |
| NM_000084.5:c.1618G>A | A540T | X | 50090199 | 50090199 | 0.542972451 |
| NM_000084.5:c.1634G>A | S545N | X | 50090215 | 50090215 | 0.871306497 |
| NM_000084.5:c.1633A>C | S545R | X | 50090214 | 50090214 | 0.835290452 |
| NM_000084.5:c.1636A>G | K546E | X | 50090217 | 50090217 | 0.916380761 |
| NM_000084.5:c.1637A>G | K546R | X | 50090218 | 50090218 | 0.842883309 |
| NM_000084.5:c.1639T>C | W547R | X | 50090220 | 50090220 | 0.955101303 |
| NM_000084.5:c.1639T>G | W547G | X | 50090220 | 50090220 | 0.870953602 |
| NM_000084.5:c.1641G>T | W547C | X | 50090222 | 50090222 | 0.920252458 |
| NM_000084.5:c.1642G>A | V548M | X | 50090223 | 50090223 | 0.859458832 |
| NM_000084.5:c.1707T>G | F569L | X | 50090288 | 50090288 | 0.160263179 |
| NM_000084.5:c.1774G>A | D592N | X | 50090355 | 50090355 | 0.831350782 |
| NM_000084.5:c.1851C>A | Y617I | X | 50090432 | 50090432 | 0.988052795 |
| NM_000084.5:c.1862C>T | P621L | X | 50090443 | 50090443 | 0.849362474 |
| NM_000084.5:c.1877G>A | R626Q | X | 50090458 | 50090458 | 0.278973026 |
| NM_000084.5:c.1906C>T | L636F | X | 50090487 | 50090487 | 0.772499423 |
| NM_000084.5:c.1958G>C | G653A | X | 50090694 | 50090694 | 0.734823708 |
| NM_000084.5:c.1970C>G | T657S | X | 50090706 | 50090706 | 0.000266954 |
| NM_000084.5:c.1978A>G | I660V | X | 50090714 | 50090714 | 0.043813815 |
| NM_000084.5:c.1988C>T | T663M | X | 50090724 | 50090724 | 0.097512363 |
| NM_000084.5:c.1987A>G | T663A | X | 50090723 | 50090723 | 0.47356078 |
| NM_000084.5:c.1991A>C | E664A | X | 50090727 | 50090727 | 0.357242475 |
| NM_000084.5:c.2051A>G | D684G | X | 50090787 | 50090787 | 0.776187776 |
| NM_000084.5:c.2083C>T | P695S | X | 50090819 | 50090819 | 0.705947893 |
| NM_000084.5:c.2095G>A | V699I | X | 50090831 | 50090831 | 0.01636133 |
| NM_000084.5:c.2108T>C | F703S | X | 50090844 | 50090844 | 0.908691186 |
| NM_000084.5:c.2111G>A | R704Q | X | 50090847 | 50090847 | 0.930699238 |
| NM_000084.5:c.2117T>C | L706P | X | 50090853 | 50090853 | 0.989355294 |
| NM_000084.5:c.2133C>G | C711W | X | 50090869 | 50090869 | 0.765485342 |
| NM_000084.5:c.2132G>C | C711S | X | 50090868 | 50090868 | 0.65494554 |
| NM_000084.5:c.2173A>G | K725E | X | 50092151 | 50092151 | 0.946014828 |

Table S4. Change in stability prediction of CLCN5 mutations using the iStable server.

| Accession number | Amino acid change | iUMutant2.0 SEQ | DDG | MUpro | Conf. Score (MUpro) | iStable | Conf. Score (iStable) |
| --- | --- | --- | --- | --- | --- | --- | --- |
| NM_000084.5:c.64T>G | W22G | D | U2.45 | D | 1 | D | 0.804497 |
| NM_000084.5:c.88C>T | R30W | D | U0.67 | D | 1 | D | 0.7693 |
| NM_000084.5:c.169G>C | G57R | D | U0.60 | I | 0.31192631 | I | 0.672511 |
| NM_000084.5:c.169G>T | G57V | D | U0.38 | I | 0.56893461 | I | 0.64004 |
| NM_000084.5:c.174G>C | W58C | D | U1.40 | D | 1 | D | 0.70598 |
| NM_000084.5:c.173G>T | W58L | D | U1.01 | D | 0.59653027 | D | 0.722181 |
| NM_000084.5:c.193G>A | G65R | null | U0.23 | I | 0.64003152 | I | 0.637044 |
| NM_000084.5:c.238C>A | H80N | D | U0.78 | D | 0.49774727 | D | 0.80963 |
| NM_000084.5:c.263G>A | G88D | D | U1.15 | D | 0.71756788 | D | 0.841911 |
| NM_000084.5:c.263G>T | G88V | D | U0.34 | D | 0.36585515 | D | 0.851451 |
| NM_000084.5:c.268T>C | C90R | null | null | null | null | D | 0.605949 |
| NM_000084.5:c.270C>G | C90W | I | 0.27 | I | 0.32330387 | I | 0.71803 |
| NM_000084.5:c.299A>G | H100R | D | null | D | 0.25831814 | D | 0.744533 |
| NM_000084.5:c.302G>A | C101Y | I | 0.15 | I | 0.37953165 | I | 0.782231 |
| NM_000084.5:c.308G>A | W103V | D | U0.62 | D | 0.84656632 | D | 0.805698 |
| NM_000084.5:c.344A>G | K115R | D | U0.10 | D | 0.10235356 | D | 0.704027 |
| NM_000084.5:c.373C>G | L125V | D | U1.40 | D | 0.29652576 | D | 0.877638 |
| NM_000084.5:c.389A>G | D130G | D | U1.06 | D | 0.42545681 | D | 0.755581 |
| NM_000084.5:c.426G>A | M142I | D | U0.68 | D | 1 | D | 0.725897 |
| NM_000084.5:c.472G>C | V158L | D | U1.48 | D | 1 | D | 0.840622 |
| NM_000084.5:c.527T>A | I176N | D | U1.88 | D | 1 | D | 0.839501 |
| NM_000084.5:c.536G>A | G179D | null | U1.01 | I | 0.32003715 | I | 0.709079 |
| NM_000084.5:c.586A>G | I196V | D | U0.40 | D | U0.29039503 | D | 0.898088 |
| NM_000084.5:c.599T>G | L200R | D | U1.42 | D | 1 | D | 0.88288 |
| NM_000084.5:c.608C>T | S203L | D | null | I | 0.48040091 | I | 0.570747 |
| NM_000084.5:c.631G>C | E211Q | D | U0.47 | I | 0.77150007 | I | 0.659621 |
| NM_000084.5:c.634G>A | G212S | D | U0.95 | D | 1 | D | 0.859642 |
| NM_000084.5:c.635G>C | G212A | D | U0.67 | D | 0.41226784 | D | 0.814111 |
| NM_000084.5:c.638C>T | P213L | D | U0.69 | D | 0.10535812 | D | 0.731483 |
| NM_000084.5:c.655T>C | C219R | D | 0.08 | D | 0.93253962 | D | 0.653982 |
| NM_000084.5:c.661T>C | C221R | D | U0.05 | D | 0.049132326 | D | 0.76009 |
| NM_000084.5:c.674T>C | L225P | D | U1.06 | D | 1 | D | 0.835892 |
| NM_000084.5:c.692A>T | K231I | D | U0.00 | D | 0.55057749 | D | 0.771845 |
| NM_000084.5:c.716G>C | R239P | D | U0.55 | D | 0.30332248 | D | 0.876516 |
| NM_000084.5:c.716G>A | R239H | D | U1.01 | D | 0.20098992 | D | 0.885756 |
| NM_000084.5:c.715C>T | R239C | null | U0.36 | D | 0.54858894 | D | 0.801565 |
| NM_000084.5:c.731C>T | S244L | null | 0.53 | I | 1 | I | 0.654263 |
| NM_000084.5:c.749G>A | G250D | null | null | null | null | Decrease | 0.547824 |
| NM_000084.5:c.748G>C | G250R | D | null | I | 0.61551481 | I | 0.563595 |
| NM_000084.5:c.751G>A | V251I | D | U0.81 | I | 0.19450503 | I | 0.643428 |
| NM_000084.5:c.766G>C | G256R | D | U0.95 | I | 0.24150045 | I | 0.611133 |
| NM_000084.5:c.779G>A | G260D | null | null | null | null | D | 0.535147 |
| NM_000084.5:c.779G>T | G260V | D | U0.63 | D | 0.64107373 | D | 0.749897 |
| NM_000084.5:c.781G>A | G261R | D | U0.79 | D | 0.2134205 | D | 0.685306 |
| NM_000084.5:c.782G>A | G261E | null | U0.77 | D | 0.41818775 | D | 0.592258 |
| NM_000084.5:c.789A>T | L263F | D | null | D | 1 | D | 0.82256 |
| NM_000084.5:c.793A>C | S265R | I | 0.08 | I | 0.98024999 | I | 0.845911 |
| NM_000084.5:c.796C>G | L266V | D | U1.54 | D | 0.22912707 | D | 0.793962 |
| NM_000084.5:c.799G>A | E267K | D | U0.84 | D | 0.7462029 | D | 0.768369 |
| NM_000084.5:c.801A>C | E267D | D | U0.23 | D | 0.55830732 | D | 0.748051 |
| NM_000084.5:c.800A>C | E267A | D | U0.72 | D | 0.54668512 | D | 0.74439 |
| NM_000084.5:c.800A>T | E267V | I | 0.42 | I | 0.24095727 | I | 0.787776 |
| NM_000084.5:c.804G>C | E268D | D | U0.24 | D | 0.54667883 | D | 0.857206 |
| NM_000084.5:c.810C>G | S270R | I | null | D | 0.024095531 | D | 0.541576 |
| NM_000084.5:c.808A>C | S270R | I | null | D | 0.024095531 | D | 0.541576 |
| NM_000084.5:c.808A>G | S270G | D | U1.01 | D | 0.92699298 | D | 0.842292 |
| NM_000084.5:c.81、3C>G | Y271V | D | U0.08 | D | 0.092546071 | D | 0.737678 |
| NM_000084.5:c.814T>A | Y272N | null | U1.07 | D | 1 | D | 0.656949 |
| NM_000084.5:c.815A>G | Y272C | D | U0.90 | D | 1 | D | 0.759584 |
| NM_000084.5:c.817T>C | F273L | null | U1.34 | D | 0.11384785 | D | 0.797309 |
| NM_000084.5:c.829A>C | T277P | null | null | null | null | D | 0.536158 |
| NM_000084.5:c.834G>C | L278F | D | U1.03 | D | 0.9046387 | D | 0.870141 |
| NM_000084.5:c.833T>C | L278S | D | U2.64 | D | 1 | D | 0.876709 |
| NM_000084.5:c.833T>G | L278W | D | U1.55 | D | 1 | D | 0.851529 |
| NM_000084.5:c.839G>C | R280P | D | U0.33 | D | 0.13338139 | D | 0.709029 |
| NM_000084.5:c.851C>T | A284V | I | 0.27 | I | 0.79969406 | I | 0.758511 |
| NM_000084.5:c.877C>T | R293C | null | U0.72 | D | 0.084504732 | I | 0.519406 |
| NM_000084.5:c.898A>G | N300D | D | U0.32 | D | 0.17055394 | D | 0.720251 |
| NM_000084.5:c.904C>T | R302C | D | null | D | 0.68421082 | D | 0.757014 |
| NM_000084.5:c.956T>G | L319R | null | null | null | null | D | 0.625585 |
| NM_000084.5:c.971T>G | L324R | D | U1.21 | D | 1 | D | 0.822265 |
| NM_000084.5:c.986G>A | G329D | D | U0.75 | I | 0.61006239 | I | 0.691084 |
| NM_000084.5:c.997G>A | G333R | D | U0.71 | I | 0.96323956 | I | 0.554634 |
| NM_000084.5:c.1013G>A | R338H | D | U1.06 | D | 0.66707978 | D | 0.756869 |
| NM_000084.5:c.1012C>T | R338C | D | U0.84 | D | 0.37473872 | D | 0.736366 |
| NM_000084.5:c.1020C>G | N340K | D | U0.74 | D | 0.41628667 | D | 0.817501 |
| NM_000084.5:c.1033C>T | R345W | D | U0.33 | D | 1 | D | 0.693521 |
| NM_000084.5:c.1034G>T | R345L | D | U0.43 | D | 0.71300955 | D | 0.74765 |
| NM_000084.5:c.1034G>A | R345Q | D | U0.48 | D | 1 | D | 0.731517 |
| NM_000084.5:c.1121A>G | N374S | D | U0.04 | I | 0.61662 | I | 0.548115 |
| NM_000084.5:c.1172A>C | D391A | D | U0.85 | D | 1 | D | 0.667205 |
| NM_000084.5:c.1184T>G | L395R | D | U1.24 | D | 0.8265548 | D | 0.857436 |
| NM_000084.5:c.1186G>C | D396H | D | U0.54 | D | 0.677139 | D | 0.744118 |
| NM_000084.5:c.1216C>T | R406C | null | U0.86 | D | 0.70088766 | D | 0.680151 |
| NM_000084.5:c.1217G>A | R406H | D | U1.35 | D | 0.66806973 | D | 0.795633 |
| NM_000084.5:c.1256C>T | P419L | D | null | D | 0.6249846 | D | 0.809659 |
| NM_000084.5:c.1264G>A | V422M | D | U1.50 | I | 0.28823538 | I | 0.556328 |
| NM_000084.5:c.1327A>G | I443V | D | U0.26 | D | 0.20499332 | D | 0.780285 |
| NM_000084.5:c.1384G>A | G462S | D | U1.59 | I | 0.14541971 | D | 0.509996 |
| NM_000084.5:c.1385G>A | G462S | D | U1.59 | I | 0.14541971 | D | 0.509996 |
| NM_000084.5:c.1385G>T | G462D | D | U1.12 | I | 1 | I | 0.535315 |
| NM_000084.5:c.1396G>C | G466R | I | U0.59 | I | 0.20351393 | I | 0.809602 |
| NM_000084.5:c.1397G>A | G466D | D | U0.98 | I | 0.48413292 | I | 0.634938 |
| NM_000084.5:c.1397G>T | G466V | D | U0.45 | I | 0.82217304 | I | 0.607573 |
| NM_000084.5:c.1403T>C | L468P | D | U1.70 | D | 0.82001428 | D | 0.8312 |
| NM_000084.5:c.1406T>C | L469P | D | U1.69 | D | 1 | D | 0.822774 |
| NM_000084.5:c.1408G>A | G470R | D | U0.64 | I | 0.12837177 | I | 0.584442 |
| NM_000084.5:c.1425G>T | Q475H | D | U0.55 | D | 0.84538451 | D | 0.777603 |
| NM_000084.5:c.1433A>G | Y478C | I | U0.79 | D | 1 | D | 0.569414 |
| NM_000084.5:c.1464C>G | S488R | I | 0.53 | I | 0.027183253 | I | 0.792522 |
| NM_000084.5:c.1475A>G | Q492R | I | 0.27 | I | 0.81454574 | I | 0.804652 |
| NM_000084.5:c.1505A>G | Y502C | I | U0.60 | I | 0.57073349 | I | 0.832469 |
| NM_000084.5:c.1510A>G | M504V | null | null | null | null | D | 0.611306 |
| NM_000084.5:c.1511T>A | M504K | D | U0.93 | D | 1 | D | 0.815149 |
| NM_000084.5:c.1514T>G | V505G | D | U2.53 | D | 0.89846357 | D | 0.845132 |
| NM_000084.5:c.1516G>A | G506R | D | U0.59 | I | 0.33004479 | I | 0.567511 |
| NM_000084.5:c.1517G>A | G506E | I | U0.55 | I | 0.22777168 | I | 0.75433 |
| NM_000084.5:c.1534G>C | G512R | D | U0.64 | D | 0.4182555 | D | 0.741132 |
| NM_000084.5:c.1535G>A | G512D | D | U1.08 | D | 0.54844656 | D | 0.737609 |
| NM_000084.5:c.1535G>T | G512V | D | U0.30 | D | 0.78996609 | D | 0.775775 |
| NM_000084.5:c.1537G>A | G513R | D | U0.59 | D | U0.064683938 | D | 0.6579 |
| NM_000084.5:c.1538G>A | G513E | I | U0.55 | I | 0.23519824 | I | 0.814198 |
| NM_000084.5:c.1547G>A | R516Q | I | U0.02 | D | 0.8161157 | D | 0.534329 |
| NM_000084.5:c.1546C>T | R516W | D | U0.07 | D | 0.37532035 | D | 0.782258 |
| NM_000084.5:c.1552A>G | T518A | D | U0.81 | D | 1 | D | 0.674146 |
| NM_000084.5:c.1556T>A | V519D | D | U1.81 | D | 0.895215 | D | 0.789646 |
| NM_000084.5:c.1558T>C | S520P | I | 0.15 | I | 0.38650018 | I | 0.649765 |
| NM_000084.5:c.1561C>T | L521F | D | U0.69 | D | 0.29046478 | D | 0.732588 |
| NM_000084.5:c.1564G>T | V522F | null | null | null | null | Decrease | 0.630687 |
| NM_000084.5:c.1571T>A | I524K | D | U1.57 | D | 1 | D | 0.857045 |
| NM_000084.5:c.1579G>C | E527Q | D | U0.40 | D | 0.53938696 | D | 0.674553 |
| NM_000084.5:c.1581A>T | E527D | D | 0 | D | 0.36252873 | D | 0.624237 |
| NM_000084.5:c.1586C>T | T529I | D | U0.24 | D | 0.00071624719 | D | 0.55797 |
| NM_000084.5:c.1588G>A | G530S | D | U1.14 | D | 0.21334904 | D | 0.921423 |
| NM_000084.5:c.1589G>T | G530V | D | U0.07 | I | 0.99971259 | D | 0.722935 |
| NM_000084.5:c.1600T>A | Y534N | D | U0.89 | D | 1 | D | 0.890741 |
| NM_000084.5:c.1618G>C | A540P | I | U0.29 | I | 0.21332197 | I | 0.802008 |
| NM_000084.5:c.1618G>A | A540T | D | U0.77 | D | 0.13569659 | D | 0.803046 |
| NM_000084.5:c.1634G>A | S545N | I | 0.28 | D | 0.55801101 | D | 0.585077 |
| NM_000084.5:c.1633A>C | S545R | I | 0.4 | D | 0.049622924 | D | 0.630019 |
| NM_000084.5:c.1636A>G | K546E | D | U0.91 | D | 0.21743869 | D | 0.761821 |
| NM_000084.5:c.1637A>G | K546R | D | U0.27 | D | 0.33794522 | D | 0.70803 |
| NM_000084.5:c.1639T>C | W547R | D | U1.02 | I | 0.208307 | D | 0.549191 |
| NM_000084.5:c.1639T>G | W547G | D | U1.97 | D | 1 | D | 0.87875 |
| NM_000084.5:c.1641G>T | W547C | D | U1.26 | D | 0.76332029 | D | 0.836763 |
| NM_000084.5:c.1642G>A | V548M | D | U0.72 | I | 0.61282329 | D | 0.556748 |
| NM_000084.5:c.1707T>G | F569L | D | U0.90 | D | 0.03298065 | D | 0.866908 |
| NM_000084.5:c.1774G>A | D592N | null | U0.43 | I | 0.006186592 | I | 0.686997 |
| NM_000084.5:c.1851C>A | Y617I | D | U0.20 | D | 0.14100089 | D | 0.693199 |
| NM_000084.5:c.1862C>T | P621L | D | U0.26 | D | 0.11918217 | D | 0.740175 |
| NM_000084.5:c.1877G>A | R626Q | D | U0.60 | D | 0.81238917 | D | 0.659103 |
| NM_000084.5:c.1906C>T | L636F | D | U0.89 | D | 1 | D | 0.906176 |
| NM_000084.5:c.1958G>C | G653A | D | U1.49 | D | 0.52132217 | D | 0.855653 |
| NM_000084.5:c.1970C>G | T657S | null | U0.58 | I | 0.75039481 | I | 0.718198 |
| NM_000084.5:c.1978A>G | I660V | null | U0.95 | D | 1 | D | 0.634026 |
| NM_000084.5:c.1988C>T | T663M | D | U0.12 | D | 0.077315297 | D | 0.660854 |
| NM_000084.5:c.1987A>G | T663A | D | U0.68 | D | 1 | D | 0.694212 |
| NM_000084.5:c.1991A>C | E664A | D | U0.62 | D | 0.7770375 | D | 0.792315 |
| NM_000084.5:c.2051A>G | D684G | D | U1.02 | D | 1 | D | 0.856324 |
| NM_000084.5:c.2083C>T | P695S | D | U1.51 | D | 1 | D | 0.825641 |
| NM_000084.5:c.2095G>A | V699I | D | U0.72 | D | 1 | D | 0.889533 |
| NM_000084.5:c.2108T>C | F703S | D | U2.04 | D | 1 | D | 0.857518 |
| NM_000084.5:c.2111G>A | R704Q | D | U0.70 | D | 0.83430751 | D | 0.784901 |
| NM_000084.5:c.2117T>C | L706P | D | U1.73 | D | 0.65579512 | D | 0.814184 |
| NM_000084.5:c.2133C>G | C711W | D | 0.05 | D | 0.6110707 | D | 0.794963 |
| NM_000084.5:c.2132G>C | C711S | D | U0.52 | D | 1 | D | 0.842294 |
| NM_000084.5:c.2173A>G | K725E | D | U0.48 | D | 0.47373034 | D | 0.812551 |

D—Decrease protein stability; I—Increase protein stability;

DDG: The change in protein folding free energy, a core quantitative indicator for measuring changes in stability. In the table, "U" denotes a negative value, and a negative value indicates that protein stability decreases after mutation (ΔΔG < 0).

Table S5. Dimer interface interactions.

**18 H-bonds**

| donor | | | | acceptor | | | | D..A dist | D-H..A dist |
| --- | --- | --- | --- | --- | --- | --- | --- | --- | --- |
| /A | SER | 27 | OG | /B | GLU | 697 | OE1 | 2.946 | N/A |
| /A | ARG | 30 | NH2 | /B | ASP | 701 | OD2 | 3.196 | N/A |
| /A | ARG | 34 | NE | /B | ALA | 577 | O | 2.637 | N/A |
| /A | ARG | 34 | NH2 | /B | PHE | 576 | O | 3.402 | N/A |
| /A | ARG | 34 | NH2 | /B | GLU | 697 | OE2 | 2.512 | N/A |
| /A | ARG | 293 | NH2 | /B | GLU | 533 | OE1 | 2.741 | N/A |
| /A | THR | 689 | OG1 | /B | THR | 689 | OG1 | 2.998 | N/A |
| /A | THR | 691 | OG1 | /B | LEU | 685 | O | 2.736 | N/A |
| /A | LYS | 705 | NZ | /B | LYS | 705 | O | 3.363 | N/A |
| /B | SER | 27 | OG | /A | GLU | 697 | OE1 | 2.919 | N/A |
| /B | ARG | 30 | NH2 | /A | ASP | 701 | OD2 | 3.108 | N/A |
| /B | ARG | 34 | NE | /A | ALA | 577 | O | 2.682 | N/A |
| /B | ARG | 34 | NH2 | /A | PHE | 576 | O | 3.418 | N/A |
| /B | ARG | 34 | NH2 | /A | GLU | 697 | OE2 | 2.384 | N/A |
| /B | ARG | 293 | NH2 | /A | GLU | 533 | OE1 | 2.79 | N/A |
| /B | THR | 689 | OG1 | /A | THR | 689 | OG1 | 2.998 | N/A |
| /B | THR | 691 | OG1 | /A | LEU | 685 | O | 2.733 | N/A |
| /B | LYS | 705 | NZ | /A | LYS | 705 | O | 3.385 | N/A |

**209 contacts**

| atom1 | | | | atom2 | | | | overlap | distance |
| --- | --- | --- | --- | --- | --- | --- | --- | --- | --- |
| /B | ILE | 535 | CG2 | /A | LEU | 286 | CD2 | 0.798 | 2.962 |
| /B | MET | 539 | SD | /A | LEU | 286 | CD1 | 0.662 | 2.988 |
| /A | MET | 539 | SD | /B | LEU | 286 | CD1 | 0.594 | 3.056 |
| /B | MET | 539 | CE | /A | PHE | 283 | CA | 0.536 | 3.224 |
| /A | MET | 539 | CE | /B | PHE | 283 | CA | 0.53 | 3.23 |
| /A | ILE | 535 | CG2 | /B | LEU | 286 | CD2 | 0.465 | 3.295 |
| /B | PHE | 688 | CZ | /A | ILE | 702 | CD1 | 0.413 | 3.227 |
| /B | MET | 539 | CE | /A | PHE | 283 | CB | 0.408 | 3.352 |
| /A | MET | 539 | CE | /B | PHE | 283 | CB | 0.38 | 3.38 |
| /A | TRP | 279 | CZ2 | /B | MET | 543 | CG | 0.377 | 3.263 |
| /A | MET | 543 | CG | /B | TRP | 279 | CZ2 | 0.365 | 3.275 |
| /B | ILE | 702 | CD1 | /A | PHE | 688 | CZ | 0.352 | 3.288 |
| /B | TRP | 279 | CE3 | /A | VAL | 519 | CG2 | 0.315 | 3.325 |
| /B | VAL | 519 | CG2 | /A | TRP | 279 | CE3 | 0.298 | 3.342 |
| /B | ARG | 34 | NH2 | /A | GLU | 697 | OE2 | 0.276 | 2.384 |
| /A | ASN | 716 | O | /B | ARG | 630 | CB | 0.24 | 3.06 |
| /A | ASN | 716 | O | /B | ARG | 630 | CD | 0.228 | 3.072 |
| /B | THR | 518 | CB | /A | TRP | 279 | CE3 | 0.224 | 3.416 |
| /B | TRP | 279 | CE3 | /A | THR | 518 | CB | 0.223 | 3.417 |
| /A | ARG | 630 | CB | /B | ASN | 716 | O | 0.203 | 3.097 |
| /A | PHE | 282 | CD1 | /B | MET | 539 | SD | 0.196 | 3.334 |
| /B | LEU | 286 | CD2 | /A | VAL | 536 | CG2 | 0.193 | 3.567 |
| /A | ARG | 30 | CD | /B | GLU | 697 | CD | 0.185 | 3.575 |
| /B | ASN | 716 | O | /A | ARG | 630 | CD | 0.172 | 3.128 |
| /B | MET | 543 | CE | /A | TRP | 58 | CZ3 | 0.168 | 3.472 |
| /B | ARG | 30 | CD | /A | GLU | 697 | CD | 0.167 | 3.593 |
| /B | GLU | 697 | OE2 | /A | ARG | 34 | NH2 | 0.148 | 2.512 |
| /A | MET | 543 | CE | /B | TRP | 58 | CZ3 | 0.147 | 3.493 |
| /A | MET | 539 | CE | /B | PHE | 283 | N | 0.146 | 3.374 |
| /B | THR | 518 | CB | /A | TRP | 279 | CD2 | 0.14 | 3.35 |
| /A | THR | 518 | CB | /B | TRP | 279 | CD2 | 0.134 | 3.356 |
| /B | MET | 539 | CE | /A | PHE | 283 | N | 0.132 | 3.388 |
| /A | ILE | 702 | CG1 | /B | PHE | 688 | CZ | 0.123 | 3.517 |
| /B | MET | 539 | SD | /A | PHE | 282 | CE1 | 0.122 | 3.408 |
| /A | MET | 539 | SD | /B | PHE | 282 | CD1 | 0.113 | 3.417 |
| /A | LEU | 266 | CD2 | /B | LEU | 266 | CD2 | 0.089 | 3.671 |
| /A | GLU | 267 | CA | /B | LEU | 275 | CD2 | 0.087 | 3.673 |
| /A | ASP | 701 | CG | /B | ARG | 30 | NH2 | 0.086 | 3.434 |
| /A | PHE | 283 | CD2 | /B | MET | 543 | SD | 0.081 | 3.449 |
| /A | LEU | 631 | O | /B | ASN | 716 | CA | 0.072 | 3.228 |
| /A | TRP | 58 | CE3 | /B | MET | 543 | CE | 0.064 | 3.576 |
| /A | LEU | 275 | CD2 | /B | GLU | 267 | CA | 0.061 | 3.699 |
| /B | PHE | 282 | CE1 | /A | MET | 539 | SD | 0.061 | 3.469 |
| /A | MET | 543 | CE | /B | TRP | 58 | CE3 | 0.052 | 3.588 |
| /A | ASN | 716 | CA | /B | LEU | 631 | O | 0.051 | 3.249 |
| /A | LYS | 705 | NZ | /B | LYS | 26 | NZ | 0.047 | 3.233 |
| /B | ILE | 702 | CG1 | /A | PHE | 688 | CZ | 0.043 | 3.597 |
| /B | PHE | 282 | CD1 | /A | MET | 539 | CE | 0.043 | 3.597 |
| /A | LEU | 275 | CD1 | /B | LYS | 573 | CE | 0.039 | 3.721 |
| /A | LYS | 26 | NZ | /B | LYS | 705 | NZ | 0.038 | 3.242 |
| /B | PHE | 283 | CD2 | /A | MET | 543 | SD | 0.032 | 3.498 |
| /B | ALA | 577 | O | /A | ARG | 34 | NE | 0.023 | 2.637 |
| /A | ARG | 30 | NH2 | /B | ASP | 701 | CG | 0.021 | 3.499 |
| /A | GLU | 697 | OE2 | /B | ARG | 34 | CZ | 0.018 | 3.012 |
| /B | LEU | 275 | CD1 | /A | LYS | 573 | CE | 0.015 | 3.745 |
| /A | ALA | 577 | O | /B | ARG | 34 | CD | 0.012 | 3.288 |
| /B | THR | 518 | C | /A | TRP | 279 | CZ3 | 0.008 | 3.362 |
| /B | TRP | 279 | CZ3 | /A | THR | 518 | C | -0.002 | 3.372 |
| /B | ALA | 577 | O | /A | ARG | 34 | CD | -0.005 | 3.305 |
| /A | ALA | 577 | O | /B | ARG | 34 | CG | -0.008 | 3.308 |
| /A | VAL | 23 | CG1 | /B | ILE | 698 | CD1 | -0.009 | 3.769 |
| /A | VAL | 519 | CG2 | /B | TRP | 279 | CA | -0.01 | 3.77 |
| /A | LYS | 705 | CD | /B | LEU | 706 | CD2 | -0.011 | 3.771 |
| /B | LYS | 705 | CD | /A | LEU | 706 | CD2 | -0.016 | 3.776 |
| /B | GLU | 697 | CB | /A | ARG | 30 | NH1 | -0.019 | 3.539 |
| /A | PHE | 688 | CE1 | /B | ILE | 702 | CD1 | -0.021 | 3.661 |
| /B | ARG | 34 | NE | /A | ALA | 577 | O | -0.022 | 2.682 |
| /B | VAL | 519 | CG2 | /A | TRP | 279 | CA | -0.022 | 3.782 |
| /A | GLU | 697 | CD | /B | ARG | 34 | NH2 | -0.024 | 3.544 |
| /A | ILE | 698 | CD1 | /B | VAL | 23 | CG1 | -0.027 | 3.787 |
| /B | MET | 539 | CE | /A | PHE | 282 | CD1 | -0.029 | 3.669 |
| /A | ASN | 716 | O | /B | ARG | 630 | CG | -0.036 | 3.336 |
| /B | GLU | 697 | OE2 | /A | ARG | 34 | CZ | -0.045 | 3.075 |
| /B | TRP | 279 | CE3 | /A | VAL | 519 | N | -0.055 | 3.455 |
| /A | ARG | 293 | NH2 | /B | GLU | 533 | CD | -0.063 | 3.583 |
| /A | ARG | 34 | CG | /B | ALA | 577 | O | -0.066 | 3.366 |
| /A | LEU | 286 | CD2 | /B | VAL | 536 | CG2 | -0.068 | 3.828 |
| /A | TRP | 279 | CE3 | /B | VAL | 519 | N | -0.069 | 3.469 |
| /B | MET | 539 | CE | /A | PHE | 283 | CD2 | -0.072 | 3.712 |
| /A | GLU | 533 | CD | /B | ARG | 293 | NH2 | -0.074 | 3.594 |
| /A | ARG | 30 | CD | /B | GLU | 697 | OE2 | -0.08 | 3.38 |
| /A | ARG | 293 | NH2 | /B | GLU | 533 | OE1 | -0.081 | 2.741 |
| /A | GLU | 697 | CB | /B | ARG | 30 | NH1 | -0.082 | 3.602 |
| /B | ASN | 716 | O | /A | ARG | 630 | CG | -0.083 | 3.383 |
| /A | ARG | 34 | NE | /B | ALA | 577 | C | -0.093 | 3.343 |
| /B | SER | 686 | CB | /A | THR | 694 | OG1 | -0.096 | 3.436 |
| /A | SER | 686 | CB | /B | THR | 694 | OG1 | -0.1 | 3.44 |
| /A | MET | 539 | CE | /B | PHE | 283 | CD2 | -0.116 | 3.756 |
| /A | ALA | 577 | C | /B | ARG | 34 | NE | -0.117 | 3.367 |
| /A | VAL | 536 | CG2 | /B | PHE | 290 | CD2 | -0.119 | 3.759 |
| /A | LEU | 685 | CD1 | /B | LEU | 693 | CD1 | -0.12 | 3.88 |
| /A | ILE | 702 | CD1 | /B | PHE | 688 | CE1 | -0.125 | 3.765 |
| /A | PHE | 526 | CE1 | /B | PHE | 526 | CE1 | -0.125 | 3.645 |
| /B | ARG | 293 | NH2 | /A | GLU | 533 | OE1 | -0.13 | 2.79 |
| /B | VAL | 536 | CG2 | /A | PHE | 290 | CD2 | -0.131 | 3.771 |
| /A | THR | 694 | CA | /B | SER | 686 | CB | -0.135 | 3.895 |
| /B | ARG | 30 | CD | /A | GLU | 697 | OE2 | -0.138 | 3.438 |
| /A | LEU | 693 | CD1 | /B | LEU | 685 | CD1 | -0.139 | 3.899 |
| /A | PHE | 282 | CE1 | /B | VAL | 522 | CG1 | -0.14 | 3.78 |
| /B | TRP | 279 | CZ3 | /A | VAL | 519 | N | -0.14 | 3.54 |
| /B | GLU | 697 | CD | /A | ARG | 34 | NH2 | -0.144 | 3.664 |
| /B | PHE | 282 | CE1 | /A | VAL | 522 | CG1 | -0.145 | 3.785 |
| /B | VAL | 519 | N | /A | TRP | 279 | CZ3 | -0.147 | 3.547 |
| /A | GLU | 697 | OE1 | /B | SER | 27 | CA | -0.151 | 3.451 |
| /A | GLU | 697 | OE1 | /B | SER | 27 | CB | -0.152 | 3.452 |
| /A | SER | 686 | CB | /B | THR | 694 | CA | -0.154 | 3.914 |
| /B | TRP | 279 | CZ3 | /A | VAL | 519 | CA | -0.155 | 3.795 |
| /A | LEU | 275 | CD1 | /B | GLU | 267 | O | -0.156 | 3.456 |
| /A | GLU | 267 | O | /B | LEU | 275 | CD1 | -0.157 | 3.457 |
| /B | MET | 539 | CE | /A | TRP | 279 | CH2 | -0.16 | 3.8 |
| /B | SER | 686 | CB | /A | THR | 694 | CG2 | -0.161 | 3.921 |
| /B | TRP | 279 | CH2 | /A | MET | 539 | CE | -0.161 | 3.801 |
| /B | PHE | 526 | CZ | /A | PHE | 526 | CZ | -0.161 | 3.681 |
| /B | VAL | 519 | CA | /A | TRP | 279 | CZ3 | -0.162 | 3.802 |
| /B | VAL | 519 | CG2 | /A | PHE | 282 | CB | -0.172 | 3.932 |
| /A | LEU | 286 | CG | /B | ILE | 535 | CG2 | -0.175 | 3.935 |
| /A | LEU | 278 | CG | /B | VAL | 519 | CG2 | -0.176 | 3.936 |
| /B | ILE | 259 | CD1 | /A | PHE | 526 | CD1 | -0.178 | 3.818 |
| /A | VAL | 519 | CG2 | /B | PHE | 282 | CB | -0.178 | 3.938 |
| /A | THR | 691 | CB | /B | LEU | 685 | O | -0.179 | 3.479 |
| /A | ARG | 30 | NH1 | /B | GLU | 697 | CG | -0.182 | 3.702 |
| /A | VAL | 519 | CG2 | /B | LEU | 278 | CG | -0.182 | 3.942 |
| /A | LEU | 685 | O | /B | THR | 691 | CB | -0.183 | 3.483 |
| /B | VAL | 523 | CG2 | /A | ILE | 259 | CD1 | -0.185 | 3.945 |
| /A | LEU | 263 | CD2 | /B | LEU | 263 | CD2 | -0.187 | 3.947 |
| /B | GLU | 697 | OE1 | /A | SER | 27 | CB | -0.188 | 3.488 |
| /A | ILE | 259 | CD1 | /B | PHE | 526 | CD1 | -0.19 | 3.83 |
| /A | SER | 27 | CA | /B | GLU | 697 | OE1 | -0.191 | 3.491 |
| /B | ILE | 535 | CG2 | /A | LEU | 286 | CD1 | -0.192 | 3.952 |
| /B | THR | 691 | OG1 | /A | SER | 686 | CA | -0.197 | 3.537 |
| /A | LYS | 705 | CD | /B | LYS | 705 | O | -0.197 | 3.497 |
| /B | SER | 686 | CB | /A | THR | 694 | CB | -0.2 | 3.96 |
| /B | MET | 543 | SD | /A | PHE | 283 | CE2 | -0.205 | 3.735 |
| /A | LYS | 705 | O | /B | LYS | 705 | CD | -0.205 | 3.505 |
| /A | SER | 686 | CB | /B | THR | 694 | CG2 | -0.206 | 3.966 |
| /A | VAL | 523 | CG2 | /B | ILE | 259 | CD1 | -0.211 | 3.971 |
| /B | LEU | 275 | CD2 | /A | GLU | 267 | CG | -0.213 | 3.973 |
| /A | SER | 686 | CB | /B | THR | 694 | CB | -0.222 | 3.982 |
| /A | THR | 691 | OG1 | /B | SER | 686 | CA | -0.223 | 3.563 |
| /A | TRP | 279 | CZ3 | /B | THR | 518 | O | -0.23 | 3.41 |
| /A | GLY | 717 | CA | /B | GLY | 717 | O | -0.23 | 3.53 |
| /A | LEU | 275 | CD2 | /B | GLU | 267 | CG | -0.234 | 3.994 |
| /A | GLY | 717 | O | /B | GLY | 717 | CA | -0.237 | 3.537 |
| /B | LEU | 685 | O | /A | THR | 691 | CG2 | -0.24 | 3.54 |
| /B | TRP | 279 | CE3 | /A | VAL | 519 | CA | -0.246 | 3.886 |
| /A | MET | 543 | SD | /B | PHE | 283 | CE2 | -0.248 | 3.778 |
| /A | TRP | 279 | CE3 | /B | THR | 518 | C | -0.25 | 3.62 |
| /B | TRP | 279 | CE3 | /A | THR | 518 | C | -0.251 | 3.621 |
| /A | LEU | 685 | O | /B | THR | 691 | OG1 | -0.253 | 2.733 |
| /B | TRP | 279 | CZ3 | /A | THR | 518 | O | -0.253 | 3.433 |
| /A | LEU | 685 | O | /B | THR | 691 | CG2 | -0.253 | 3.553 |
| /A | TRP | 279 | CE3 | /B | VAL | 519 | CA | -0.255 | 3.895 |
| /B | TRP | 279 | CZ3 | /A | MET | 539 | CE | -0.255 | 3.895 |
| /B | ARG | 30 | NH1 | /A | GLU | 697 | CG | -0.255 | 3.775 |
| /B | LEU | 685 | O | /A | THR | 691 | OG1 | -0.256 | 2.736 |
| /B | LEU | 275 | CD1 | /A | LYS | 573 | CG | -0.259 | 4.019 |
| /A | TRP | 279 | CH2 | /B | VAL | 522 | CG2 | -0.263 | 3.903 |
| /A | ASN | 716 | CB | /B | VAL | 624 | CG2 | -0.263 | 4.023 |
| /A | TRP | 279 | CZ3 | /B | MET | 539 | CE | -0.264 | 3.904 |
| /B | THR | 518 | CB | /A | TRP | 279 | CZ3 | -0.264 | 3.904 |
| /A | LEU | 275 | CD1 | /B | LYS | 573 | CG | -0.266 | 4.026 |
| /A | VAL | 522 | CG2 | /B | TRP | 279 | CH2 | -0.266 | 3.906 |
| /B | TRP | 279 | CZ3 | /A | THR | 518 | CB | -0.27 | 3.91 |
| /A | TRP | 279 | CG | /B | THR | 518 | CB | -0.277 | 3.767 |
| /A | THR | 518 | CB | /B | TRP | 279 | CG | -0.284 | 3.774 |
| /B | ARG | 34 | NH1 | /A | GLU | 697 | OE2 | -0.288 | 2.948 |
| /B | THR | 518 | CB | /A | TRP | 279 | CE2 | -0.293 | 3.783 |
| /A | ARG | 34 | NH2 | /B | HIS | 578 | O | -0.296 | 2.956 |
| /A | TRP | 314 | CZ2 | /B | PHE | 290 | CE2 | -0.301 | 3.821 |
| /A | THR | 518 | CB | /B | TRP | 279 | CE2 | -0.305 | 3.795 |
| /A | VAL | 624 | CG2 | /B | ASN | 716 | CB | -0.308 | 4.068 |
| /B | MET | 543 | CE | /A | TRP | 58 | CH2 | -0.309 | 3.949 |
| /B | GLU | 697 | OE2 | /A | ARG | 34 | NH1 | -0.311 | 2.971 |
| /A | MET | 543 | CE | /B | TRP | 58 | CH2 | -0.313 | 3.953 |
| /A | PHE | 290 | CE2 | /B | TRP | 314 | CZ2 | -0.316 | 3.836 |
| /B | VAL | 624 | CG2 | /A | ASN | 716 | ND2 | -0.317 | 3.837 |
| /B | THR | 518 | CA | /A | TRP | 279 | CZ3 | -0.34 | 3.98 |
| /A | ILE | 259 | CG2 | /B | VAL | 523 | CG2 | -0.342 | 4.102 |
| /A | VAL | 523 | CG2 | /B | ILE | 259 | CG2 | -0.345 | 4.105 |
| /B | MET | 543 | CE | /A | PHE | 283 | CD2 | -0.347 | 3.987 |
| /A | THR | 518 | CA | /B | TRP | 279 | CZ3 | -0.35 | 3.99 |
| /A | TRP | 279 | CE3 | /B | THR | 518 | CA | -0.358 | 3.998 |
| /A | PHE | 282 | CE1 | /B | VAL | 522 | CB | -0.359 | 3.999 |
| /A | PHE | 688 | CE1 | /B | ILE | 702 | CG1 | -0.359 | 3.999 |
| /B | PHE | 526 | CE1 | /A | PHE | 526 | CZ | -0.36 | 3.88 |
| /A | GLU | 697 | CD | /B | ARG | 34 | NH1 | -0.362 | 3.882 |
| /A | THR | 518 | CA | /B | TRP | 279 | CE3 | -0.364 | 4.004 |
| /A | PHE | 526 | CE1 | /B | PHE | 526 | CZ | -0.364 | 3.884 |
| /A | TRP | 547 | CZ2 | /B | TRP | 58 | NE1 | -0.368 | 3.768 |
| /A | ILE | 535 | CG2 | /B | LEU | 286 | CG | -0.37 | 4.13 |
| /A | GLU | 697 | CB | /B | ARG | 30 | CD | -0.37 | 4.13 |
| /B | MET | 543 | CE | /A | PHE | 283 | CE2 | -0.374 | 4.014 |
| /B | TRP | 279 | CZ3 | /A | VAL | 519 | CG2 | -0.374 | 4.014 |
| /A | VAL | 624 | CG2 | /B | ASN | 716 | ND2 | -0.376 | 3.896 |
| /B | VAL | 519 | CG2 | /A | LEU | 278 | CD2 | -0.379 | 4.139 |
| /B | TRP | 547 | CZ2 | /A | TRP | 58 | NE1 | -0.379 | 3.779 |
| /B | TRP | 547 | CZ2 | /A | TRP | 58 | CE2 | -0.38 | 3.75 |
| /A | LEU | 286 | CD1 | /B | MET | 539 | CG | -0.38 | 4.14 |
| /B | GLU | 697 | CD | /A | ARG | 34 | NH1 | -0.38 | 3.9 |
| /A | ARG | 34 | NH2 | /B | HIS | 578 | C | -0.38 | 3.63 |
| /A | LEU | 286 | CD1 | /B | MET | 539 | CB | -0.383 | 4.143 |
| /A | ARG | 30 | CD | /B | GLU | 697 | CB | -0.387 | 4.147 |
| /A | VAL | 522 | CB | /B | PHE | 282 | CE1 | -0.387 | 4.027 |
| /A | VAL | 522 | CG1 | /B | PHE | 282 | CZ | -0.387 | 4.027 |
| /B | PHE | 283 | CD2 | /A | MET | 543 | CE | -0.388 | 4.028 |
| /B | VAL | 519 | CG2 | /A | TRP | 279 | CZ3 | -0.39 | 4.03 |
| /A | VAL | 519 | CG2 | /B | LEU | 278 | CD2 | -0.397 | 4.157 |
| /A | TRP | 58 | CD1 | /B | TRP | 547 | CZ2 | -0.398 | 3.918 |
| /A | TRP | 547 | CZ2 | /B | TRP | 58 | CE2 | -0.398 | 3.768 |

**2 clashes**

| atom1 | | | | atom2 | | | | overlap | distance |
| --- | --- | --- | --- | --- | --- | --- | --- | --- | --- |
| /A | LEU | 286 | CD2 | /B | ILE | 535 | CG2 | 0.798 | 2.962 |
| /A | LEU | 286 | CD1 | /B | MET | 539 | SD | 0.662 | 2.988 |

Table S6. Biophysical characterization of CLCN5 mutations using the Align GVGD server.

| Accession Number | Amino acid change | GV | GD | Prediction |
| --- | --- | --- | --- | --- |
| CM983857 | W22G | 0 | 183.79 | Class C65 |
| CM187918 | R30W | 0 | 101.29 | Class C65 |
| CM1617184 | G57R | 0 | 125.13 | Class C65 |
| CM970315 | G57V | 0 | 108.79 | Class C65 |
| CM097568 | W58C | 0 | 214.36 | Class C65 |
| CM095944 | W58L | 0 | 60.98 | Class C55 |
| CM1314273 | G65R | 0 | 125.13 | Class C65 |
| NM_000084.5:c.238C>A | H80N | 0 | 68.35 | Class C65 |
| CM066011 | G88D | 0 | 93.77 | Class C65 |
| CM139666 | G88V | 0 | 108.79 | Class C65 |
| NM_000084.5:c.268T>C | C90R | 0 | 179.53 | Class C65 |
| CM139667 | C90W | 0 | 214.36 | Class C65 |
| CM1414749 | H100R | 0 | 28.82 | Class C25 |
| CM082570 | C101Y | 0 | 193.72 | Class C65 |
| CM139669 | W103R | 0 | 101.29 | Class C65 |
| NM_000084.5:c.344A>G | K115R | 0 | 26 | Class C25 |
| NM_000084.5:c.373C>G | L125V | 0 | 31.78 | Class C25 |
| CM1414345 | D130G | 0 | 93.77 | Class C65 |
| NM_000084.5:c.426G>A | M142I | 0 | 10.12 | Class C0 |
| NM_000084.5:c.472G>C | V158L | 0 | 31.78 | Class C25 |
| CM139671 | I176N | 0 | 148.91 | Class C65 |
| CM097071 | G179D | 0 | 93.77 | Class C65 |
| NM_000084.5:c.586A>G | I196V | 0 | 29.61 | Class C25 |
| NM_000084.5:c.599T>G | L200R | 0 | 101.88 | Class C65 |
| CM097072 | S203L | 0 | 144.08 | Class C65 |
| CM139673 | E211Q | 0 | 29.27 | Class C25 |
| CM139674 | G212A | 0 | 60 | Class C55 |
| CM097073 | G212S | 0 | 55.27 | Class C55 |
| CM139675 | P213L | 0 | 97.78 | Class C65 |
| CM071612 | C219R | 0 | 179.53 | Class C65 |
| NM_000084.5:c.661T>C | C221R | 0 | 179.53 | Class C65 |
| CM077238 | L225P | 0 | 97.78 | Class C65 |
| CM157776 | K231I | 0 | 101.61 | Class C65 |
| CM157777 | R239C | 0 | 179.53 | Class C65 |
| NM_000084.5:c.716G>A | R239H | 0 | 28.82 | Class C25 |
| NM_000084.5:c.715C>T | R239P | 0 | 102.71 | Class C65 |
| CM960309 | S244L | 0 | 144.08 | Class C65 |
| NM_000084.5:c.749G>A | G250D | 0 | 93.77 | Class C65 |
| CM157778 | G250R | 0 | 125.13 | Class C65 |
| NM_000084.5:c.751G>A | V251I | 0 | 29.61 | Class C25 |
| NM_000084.5:c.766G>C | G256R | 0 | 125.13 | Class C65 |
| NM_000084.5:c.779G>A | G260D | 0 | 93.77 | Class C65 |
| CM062520 | G260V | 0 | 108.79 | Class C65 |
| CM095945 | G261E | 0 | 97.85 | Class C65 |
| CM104899 | G261R | 0 | 125.13 | Class C65 |
| CM166359 | L263F | 0 | 21.82 | Class C15 |
| CM166360 | S265R | 0 | 109.21 | Class C65 |
| CM139676 | L266V | 0 | 31.78 | Class C25 |
| CM153737 | E267A | 0 | 106.71 | Class C65 |
| CM157779 | E267D | 0 | 44.6 | Class C35 |
| CM044216 | E267A | 0 | 56.87 | Class C55 |
| CM185956 | E267V | 0 | 121.33 | Class C65 |
| CM1516353 | E268D | 0 | 44.6 | Class C35 |
| CM044217 | S270G | 0 | 55.27 | Class C55 |
| CM980369 | S270R | 0 | 109.21 | Class C65 |
| CM066756 | Y272C | 0 | 193.72 | Class C65 |
| CM139677 | Y272N | 0 | 142.23 | Class C65 |
| CM071613 | F273L | 0 | 21.82 | Class C15 |
| NM_000084.5:c.829A>C | T277P | 0 | 37.56 | Class C35 |
| CM980370 | L278F | 0 | 21.82 | Class C15 |
| CM166356 | L278S | 0 | 144.08 | Class C65 |
| CM122880 | L278W | 0 | 60.98 | Class C55 |
| CM970316 | R280P | 0 | 102.71 | Class C65 |
| NM_000084.5:c.851C>T | A284V | 0 | 64.43 | Class C55 |
| NM_000084.5:c.877C>T | R293C | 0 | 179.53 | Class C65 |
| NM_000084.5:c.898A>G | N300D | 0 | 23.01 | Class C15 |
| NM_000084.5:c.904C>T | R302C | 0 | 179.53 | Class C65 |
| NM_000084.5:c.956T>G | L319R | 0 | 101.88 | Class C65 |
| CM052170 | L324R | 0 | 101.88 | Class C65 |
| CM122879 | G329D | 0 | 93.77 | Class C65 |
| CM077523 | G333R | 0 | 125.13 | Class C65 |
| NM_000084.5:c.1012C>T | R338C | 0 | 179.53 | Class C65 |
| NM_000084.5:c.1013G>A | R338H | 0 | 28.82 | Class C25 |
| CM066755 | N340K | 0 | 93.88 | Class C65 |
| NM_000084.5:c.1034G>T | R345L | 0 | 101.88 | Class C65 |
| NM_000084.5:c.1034G>A | R345Q | 0 | 42.81 | Class C35 |
| CM165162 | R345W | 0 | 101.29 | Class C65 |
| NM_000084.5:c.1121A>G | N374S | 0 | 46.24 | Class C45 |
| NM_000084.5:c.1172A>C | D391A | 0 | 125.75 | Class C65 |
| NM_000084.5:c.1184T>G | L395R | 0 | 101.88 | Class C65 |
| NM_000084.5:c.1186G>C | D396H | 0 | 81.24 | Class C65 |
| NM_000084.5:c.1216C>T | R406C | 0 | 179.53 | Class C65 |
| NM_000084.5:c.1217G>A | R406H | 0 | 28.82 | Class C25 |
| NM_000084.5:c.1256C>T | P419L | 0 | 97.78 | Class C65 |
| NM_000084.5:c.1264G>A | V422M | 0 | 21.52 | Class C15 |
| NM_000084.5:c.1327A>G | I443V | 0 | 29.61 | Class C25 |
| CM052172 | G462D | 0 | 93.77 | Class C65 |
| CM044218 | G462S | 0 | 55.27 | Class C55 |
| CM1314274 | G466D | 0 | 93.77 | Class C65 |
| CM125772 | G466R | 0 | 125.13 | Class C65 |
| NM_000084.5:c.1397G>T | G466V | 0 | 108.79 | Class C65 |
| CM139679 | L468P | 0 | 97.78 | Class C65 |
| CM097074 | L469P | 0 | 97.78 | Class C65 |
| CM157780 | G470R | 0 | 125.13 | Class C65 |
| NM_000084.5:c.1425G>T | Q475H | 0 | 24.08 | Class C15 |
| NM_000084.5:c.1433A>G | Y478C | 0 | 193.72 | Class C65 |
| NM_000084.5:c.1464C>G | S488R | 0 | 109.21 | Class C65 |
| NM_000084.5:c.1475A>G | Q492R | 0 | 42.81 | Class C35 |
| CM139682 | Y502C | 0 | 193.72 | Class C65 |
| NM_000084.5:c.1510A>G | M504V | 0 | 21.52 | Class C15 |
| CM097570 | M504K | 0 | 94.49 | Class C65 |
| CM109654 | V505G | 0 | 108.79 | Class C65 |
| CM960311 | G506E | 0 | 97.85 | Class C65 |
| CM139683 | G506R | 0 | 125.13 | Class C65 |
| CM095946 | G512D | 0 | 93.77 | Class C65 |
| CM970318 | G512R | 0 | 125.13 | Class C65 |
| NM_000084.5:c.1535G>T | G512V | 0 | 108.79 | Class C65 |
| CM973255 | G513E | 0 | 97.85 | Class C65 |
| CM044219 | G513R | 0 | 125.13 | Class C65 |
| CM139684 | R516Q | 0 | 42.81 | Class C35 |
| CM973256 | R516W | 0 | 101.29 | Class C65 |
| CM157781 | T518A | 0 | 58.02 | Class C55 |
| CM095947 | V519D | 0 | 152.01 | Class C65 |
| CM960312 | S520P | 0 | 73.35 | Class C65 |
| CM146435 | L521F | 0 | 21.82 | Class C15 |
| NM_000084.5:c.1564G>T | V522F | 0 | 49.94 | Class C45 |
| CM010188 | I524K | 0 | 101.61 | Class C65 |
| CM1617183 | E527D | 0 | 44.6 | Class C35 |
| CM970319 | E527Q | 0 | 29.27 | Class C25 |
| NM_000084.5:c.1586C>T | T529I | 0 | 89.28 | Class C65 |
| CM157782 | G530S | 0 | 55.27 | Class C55 |
| CM1718625 | G530V | 0 | 108.79 | Class C65 |
| NM_000084.5:c.1600T>A | Y534N | 0 | 142.23 | Class C65 |
| CM1613493 | A540P | 0 | 26.87 | Class C25 |
| NM_000084.5:c.1618G>A | A540T | 0 | 58.02 | Class C55 |
| CM044220 | S545N | 0 | 46.24 | Class C45 |
| CM1718627 | S545R | 0 | 109.21 | Class C65 |
| CM044221 | K546E | 0 | 56.87 | Class C55 |
| NM_000084.5:c.1637A>G | K546R | 0 | 26 | Class C25 |
| NM_000084.5:c.1641G>T | W547C | 0 | 214.36 | Class C65 |
| CM071609 | W547G | 0 | 183.79 | Class C65 |
| CM095948 | W547R | 0 | 101.29 | Class C65 |
| NM_000084.5:c.1642G>A | V548M | 0 | 21.52 | Class C15 |
| NM_000084.5:c.1707T>G | F569L | 0 | 21.82 | Class C15 |
| NM_000084.5:c.1774G>A | D592N | 0 | 23.01 | Class C15 |
| CM004240 | Y617I | 0 | 33.05 | Class C25 |
| CM095949 | P621L | 0 | 97.78 | Class C65 |
| NM_000084.5:c.1877G>A | R626Q | 0 | 42.81 | Class C35 |
| NM_000084.5:c.1906C>T | L636F | 0 | 21.82 | Class C15 |
| NM_000084.5:c.1958G>C | G653A | 0 | 60 | Class C55 |
| CM044222 | T657S | 0 | 57.75 | Class C55 |
| NM_000084.5:c.1978A>G | I660V | 0 | 29.61 | Class C25 |
| NM_000084.5:c.1987A>G | T663A | 0 | 58.02 | Class C55 |
| NM_000084.5:c.1988C>T | T663M | 0 | 81.04 | Class C65 |
| NM_000084.5:c.1991A>C | E664A | 0 | 106.71 | Class C65 |
| NM_000084.5:c.2051A>G | D684G | 0 | 93.77 | Class C65 |
| NM_000084.5:c.2083C>T | P695S | 0 | 73.35 | Class C65 |
| NM_000084.5:c.2095G>A | V699I | 0 | 29.61 | Class C25 |
| CM139686 | F703S | 0 | 154.81 | Class C65 |
| NM_000084.5:c.2111G>A | R704Q | 0 | 42.81 | Class C35 |
| CM139687 | L706P | 0 | 97.78 | Class C65 |
| NM_000084.5:c.2132G>C | C711S | 0 | 111.67 | Class C65 |
| CM139688 | C711W | 0 | 214.36 | Class C65 |
| CM066010 | K725E | 0 | 56.87 | Class C55 |

The following classifiers, ordered from most likely to interfere with function to least likely, were used:

GD>=65+Tan(10)x(GV^2.5) => Class C65 <=> most likely

GD>=55+Tan(10)x(GV^2.0) => Class C55

GD>=45+Tan(15)x(GV^1.7) => Class C45

GD>=35+Tan(50)x(GV^1.1) => Class C35

GD>=25+Tan(55)x(GV^0.95) => Class C25

GD>=15+Tan(75)x(GV^0.6) => Class C15

Else (GD<15+Tan(75)x(GV^0.6)) => Class C0 <=> less likely

Table S7. Phenotypic effect prediction of pathogenic CLCN5 mutations using the SNPeffect server.

| UniProt ID | Mutation | dTANGO | dWALTZ | dLIMBO | dFoldX |
| --- | --- | --- | --- | --- | --- |
| CLCN5_HUMAN | **W22G** | Does not affect the aggregation tendency of your protein | Does not affect the amyloid propensity of your protein | Does not affect the chaperone binding tendency of your protein | N.A. |
| CLCN5_HUMAN | **W58C** | Decreases the aggregation tendency of your protein | Does not affect the amyloid propensity of your protein | Does not affect the chaperone binding tendency of your protein | N.A. |
| CLCN5_HUMAN | **G88D** | Does not affect the aggregation tendency of your protein | Does not affect the amyloid propensity of your protein | Does not affect the chaperone binding tendency of your protein | N.A. |
| CLCN5_HUMAN | **G88V** | Increases the aggregation tendency of your protein | Does not affect the amyloid propensity of your protein | Does not affect the chaperone binding tendency of your protein | N.A. |
| CLCN5_HUMAN | **L200R** | Decreases the aggregation tendency of your protein | Does not affect the amyloid propensity of your protein | Does not affect the chaperone binding tendency of your protein | N.A. |
| CLCN5_HUMAN | **P213L** | Increases the aggregation tendency of your protein | Does not affect the amyloid propensity of your protein | Does not affect the chaperone binding tendency of your protein | N.A. |
| CLCN5_HUMAN | **C219R** | Does not affect the aggregation tendency of your protein | Does not affect the amyloid propensity of your protein | Does not affect the chaperone binding tendency of your protein | N.A. |
| CLCN5_HUMAN | **C221R** | Does not affect the aggregation tendency of your protein | Does not affect the amyloid propensity of your protein | Does not affect the chaperone binding tendency of your protein | N.A. |
| CLCN5_HUMAN | **R239P** | Does not affect the aggregation tendency of your protein | Does not affect the amyloid propensity of your protein | Does not affect the chaperone binding tendency of your protein | N.A. |
| CLCN5_HUMAN | **G260V** | Increases the aggregation tendency of your protein | Decreases the amyloid propensity of your protein | Does not affect the chaperone binding tendency of your protein | N.A. |
| CLCN5_HUMAN | **G261R** | Increases the aggregation tendency of your protein | Decreases the amyloid propensity of your protein | Does not affect the chaperone binding tendency of your protein | N.A. |
| CLCN5_HUMAN | **E267A** | Increases the aggregation tendency of your protein | Does not affect the amyloid propensity of your protein | Does not affect the chaperone binding tendency of your protein | N.A. |
| CLCN5_HUMAN | **R280P** | Does not affect the aggregation tendency of your protein | Does not affect the amyloid propensity of your protein | Does not affect the chaperone binding tendency of your protein | N.A. |
| CLCN5_HUMAN | **L324R** | Decreases the aggregation tendency of your protein | Does not affect the amyloid propensity of your protein | Does not affect the chaperone binding tendency of your protein | N.A. |
| CLCN5_HUMAN | **R338C** | Increases the aggregation tendency of your protein | Does not affect the amyloid propensity of your protein | Does not affect the chaperone binding tendency of your protein | N.A. |
| CLCN5_HUMAN | **L468P** | Does not affect the aggregation tendency of your protein | Does not affect the amyloid propensity of your protein | Does not affect the chaperone binding tendency of your protein | N.A. |
| CLCN5_HUMAN | **L469P** | Does not affect the aggregation tendency of your protein | Does not affect the amyloid propensity of your protein | Does not affect the chaperone binding tendency of your protein | N.A. |
| CLCN5_HUMAN | **M504K** | Decreases the aggregation tendency of your protein | Does not affect the amyloid propensity of your protein | Does not affect the chaperone binding tendency of your protein | N.A. |
| CLCN5_HUMAN | **V505G** | Decreases the aggregation tendency of your protein | Does not affect the amyloid propensity of your protein | Does not affect the chaperone binding tendency of your protein | N.A. |
| CLCN5_HUMAN | **G512R** | Does not affect the aggregation tendency of your protein | Does not affect the amyloid propensity of your protein | Does not affect the chaperone binding tendency of your protein | N.A. |
| CLCN5_HUMAN | **G512V** | Does not affect the aggregation tendency of your protein | Does not affect the amyloid propensity of your protein | Does not affect the chaperone binding tendency of your protein | N.A. |
| CLCN5_HUMAN | **R516W** | Increases the aggregation tendency of your protein | Increases the amyloid propensity of your protein | Does not affect the chaperone binding tendency of your protein | N.A. |
| CLCN5_HUMAN | **V519D** | Does not affect the aggregation tendency of your protein | Increases the amyloid propensity of your protein | Does not affect the chaperone binding tendency of your protein | N.A. |
| CLCN5_HUMAN | **I524K** | Does not affect the aggregation tendency of your protein | Increases the amyloid propensity of your protein | Does not affect the chaperone binding tendency of your protein | N.A. |
| CLCN5_HUMAN | **T529I** | Does not affect the aggregation tendency of your protein | Decreases the amyloid propensity of your protein | Does not affect the chaperone binding tendency of your protein | N.A. |
| CLCN5_HUMAN | **P621L** | Increases the aggregation tendency of your protein | Does not affect the amyloid propensity of your protein | Does not affect the chaperone binding tendency of your protein | Reduces the protein stability |
| CLCN5_HUMAN | **D684G** | Does not affect the aggregation tendency of your protein | Does not affect the amyloid propensity of your protein | Does not affect the chaperone binding tendency of your protein | Reduces the protein stability |
| CLCN5_HUMAN | **F703S** | Decreases the aggregation tendency of your protein | Does not affect the amyloid propensity of your protein | Does not affect the chaperone binding tendency of your protein | Reduces the protein stability |
| CLCN5_HUMAN | **L706P** | Does not affect the aggregation tendency of your protein | Does not affect the amyloid propensity of your protein | Does not affect the chaperone binding tendency of your protein | Reduces the protein stability |
| CLCN5_HUMAN | **C711W** | Does not affect the aggregation tendency of your protein | Does not affect the amyloid propensity of your protein | Does not affect the chaperone binding tendency of your protein | Severely reduces the protein stability |

Table S8. Clinical patient data from individuals with CLCN5 gene mutations in China

| Patient | protein mutation | Age of onset | Serum creatinine (umol/L) | urinary erthrocyteHP | Serum albumin (g/L) | urine mALb/Ucr (mg/g) | urine α1MG/Ucr(mg/g) | UPE (mg/kg/24 h) | Proteinuria (mg/kg/24 h) | Proteinuria/Creatinine (mg/mg) | UC/Ucr (mg/mg) | Urine Calcium (mmol/24 h) | Serium creatine kinase (U/L) | onset symptom | first eGFR (ml/min) | CKD | Renal ultrasound | Renal biopsy |
| --- | --- | --- | --- | --- | --- | --- | --- | --- | --- | --- | --- | --- | --- | --- | --- | --- | --- | --- |
| 1 | V522F | 4 | 43.5 | 0.7 | 42 | 414.7 | 379.1 |  |  | 2.76 | 0.424 | 5.69 | 83 | 1 | 115 | 1 | 1 |  |
| 2 | S244L | 11 | 41.8 | 0.59 | 47.5 | 291.3 | 384.3 |  | 2.26 | 2.09 | 0.954 | 5.4 | 130 | 1 | 118 | 1 | 3 |  |
| 3 | G530V | 4.5 | 23 | 1.55 | 49.7 | 663 | 633.5 |  |  | 3.27 | 0.45 | 9.13 | 201 | 1 | 177 | 1 | 1 | MCD |
| 4 | C90R | 0.5 | 20 | 0.14 | 40.3 | 1084.6 | 513.6 |  | 8.5 | 6.46 | 0.28 |  | 72 | 1 | 122 | 1 | 1 | MCD |
| 5 | G250D | 2 | 29 | 1.4 | 47.1 | 409.9 | 358.3 |  |  | 2.44 | 0.041 | 0.588 | 320 | 2 | 123.3 | 1 | 1 | MCD |
| 6 | G512V | 3 | 32 | 0.74 | 43.2 | 156.8 | 326.3 |  |  | 2.07 | 0.509 |  | 136 | 1 |  |  | 1 |  |
| 7 | M504V | 2 | 22.3 | 0.05 | 45.45 | 287.2 | 453.7 |  |  | 2.67 | 0.263 |  |  | 1 |  |  | 1 | MCD |
| 8 | Y272C | 0 | 19 | 18.25 | 25.3 | 367 | 1231.5 |  |  | 5.34 |  |  | 106 | 1 |  |  | 4 |  |
| 9 | G466R | 9 | 43.6 | 2.36 | 43.2 |  |  | 27.33 |  | 0.15 | 0.34 | 4.07 | 126 | 1 | 304.34 | 1 | 4 |  |
| 10 | G466D | 5 | 58 | 5.18 | 44.9 |  |  | 38.14 |  |  | 0.37 | 20.3 | 230 |  | 129.2 | 1 |  | MCD |
| 11 | G250D | 3 |  |  | 45.6 |  |  | 40.14 | 5.8 |  | 0.14 | 3.92 |  | 1 | 104.14 | 1 | 1 | MCD |
| 12 | T277P | 14 |  | 21.6 | 32.5 |  |  |  |  |  |  |  | 51 | 1 | 108 | 1 |  |  |
| 13 | G513R | 9 | 33 | 5.4 | 45.2 | 313.59 | 364.28 |  | 49.3 | 0.48 | 1.9 | 3.88 |  | 2 |  |  | 1 |  |
| 14 | R516W | 8.5 | 35 | 3.7 | 44.7 | 183.2 | 255.95 | 40.9 | 40.9 | 1.77 | 0.49 | 0.25 | 102 | 1 | 147 | 1 | 1 | FSGS |
| 15 | L319R | 10.5 | 38 | 0.5 | 42 | 318.77 | 449.92 | 42 | 42 | 4.18 |  |  | 89 | 1 |  |  | 1 | MCD |
| 16 | R516W | 3 | 49.1 | 0.2 | 46.9 | 253.53 | 415.33 | 51.1 | 51.1 | 3.02 | 0.539 | 0.19 | 180 | 1 | 104 | 1 | 1 |  |
| 17 | G470R | 4.5 | 30.6 | 2.4 | 41.7 | 188 | 360.32 | 20.37 | 20.37 | 1.1 | 0.35 | 0.14 | 30.6 | 1 | 134 | 1 | 1 |  |
| 18 | G512R | 3.2 | 33.5 | 0 | 40.6 | 477.29 | 583.85 | 54.6 | 54.6 | 2.361881254 |  | 4.6 | 206 | 1 | 228 | 1 | 1 |  |
| 19 | M504V | 4 | 46.1 | 0 | 36.1 | 264.8 | 456.3 | 35.3 | 35.3 | 1.989660073 |  | 5.68 | 65 | 1 | 204 | 1 | 4 | MCD |
| 20 | G88N | 2 | 35.3 | 0 | 38.1 | 609.4 | 1350.45 | 147.5 | 147.5 | 5.888653846 |  | 4.1 | NA | 1 | 200 | 1 | 1 |  |
| 21 | G57V | 1.8 | 26.4 | 14 | 37.8 | 843.59 | 987.55 | 92.6 | 92.6 | 3.775169501 |  | 3.33 | NA | 2 | 194 | 1 | 4 |  |
| 22 | G260D | 4.8 | 51 | 20 | 24.2 | 468.42 | 688.91 | 47.7 | 47.7 | 2.223 |  | 5.99 | NA | 1 | 101 | 1 | 4 | MCD |

Renal ultrasound: 1 (normal), 2 (one kidney), 3 (one kidney, stone or two kidney), 4 (two kidney stone).

onset symptom: 1 (proteinuria), 2 (edema，Proteinuria , Hematuria, kideny stone).

CKD (Chronic Kidney Disease staging): CKD Stage 1 —— Normal or mildly decreased renal function.

Renal biopsy: MCD: Minimal Change Disease; FSGS: Focal Segmental Glomerulosclerosis.

Table S9. Pathogenicity predictions of clinical patient with CLCN5 gene mutations in China

| protein mutation | PredictSNP | MAPP | PhDUSNP | PolyPhenU1 | PolyPhenU2 | SIFT | SNAP | PANTHER | GVGD Prediction | MAGPIE prediction | iStable |
| --- | --- | --- | --- | --- | --- | --- | --- | --- | --- | --- | --- |
| G57V | N | N | D | D | N | D | N | U | D | D | Increase |
| G88D | D | D | D | D | D | D | D | U | D | D | Decrease |
| C90R | D | D | D | D | D | D | D | U | D | D | Decrease |
| S244L | D | D | D | D | D | D | D | U | D | D | Increase |
| G250D | D | D | D | D | D | D | D | U | D | D | Decrease |
| G260D | D | D | D | D | D | D | D | U | D | N | Decrease |
| Y272C | D | N | D | D | D | D | D | U | D | D | Decrease |
| T277P | D | D | D | D | D | D | D | D | N | D | Decrease |
| L319R | D | D | D | D | D | D | D | U | D | N | Decrease |
| G466R | D | D | D | D | D | D | D | U | D | D | Increase |
| G466D | D | D | D | D | D | D | D | U | D | D | Increase |
| G470R | D | D | D | D | D | D | D | U | D | D | Increase |
| M504V | N | D | D | N | N | D | N | U | N | N | Decrease |
| G512V | D | D | D | D | D | D | D | U | D | D | Decrease |
| G512R | D | D | D | D | D | D | D | U | D | D | Decrease |
| G513R | D | D | D | D | D | D | D | U | D | D | Decrease |
| R516W | D | D | D | D | D | D | D | U | D | D | Decrease |
| V522F | D | D | D | D | D | D | D | U | D | N | Decrease |
| G530V | D | D | D | D | D | D | D | U | D | D | Decrease |

D—Deleterious, N—Neutral, U—Unknown; Decrease: decrease protein stability; Increase: increase protein stability.
